# Musashi-1: An Example of How Polyalanine Tracts Contribute to Self-Association in the Intrinsically Disordered Regions of RNA-Binding Proteins

**DOI:** 10.3390/ijms21072289

**Published:** 2020-03-26

**Authors:** Tsai-Chen Chen, Jie-rong Huang

**Affiliations:** 1 Institute of Biochemistry and Molecular Biology, National Yang-Ming University, No. 155 Section 2 Li-nong Street, Taipei 11221, Taiwan; 2 Institute of Biomedical Informatics, National Yang-Ming University, No. 155 Section 2 Li-nong Street, Taipei 11221, Taiwan

**Keywords:** RNA-binding proteins, intrinsically disordered proteins, polyalanine, liquid–liquid phase separation, self-association

## Abstract

RNA-binding proteins (RBPs) have intrinsically disordered regions (IDRs) whose biophysical properties have yet to be explored to the same extent as those of the folded RNA interacting domains. These IDRs are essential to the formation of biomolecular condensates, such as stress and RNA granules, but dysregulated assembly can be pathological. Because of their structural heterogeneity, IDRs are best studied by NMR spectroscopy. In this study, we used NMR spectroscopy to investigate the structural propensity and self-association of the IDR of the RBP Musashi-1. We identified two transient α-helical regions (residues ~208–218 and ~270–284 in the IDR, the latter with a polyalanine tract). Strong NMR line broadening in these regions and circular dichroism and micrography data suggest that the two α-helical elements and the hydrophobic residues in between may contribute to the formation of oligomers found in stress granules and implicated in Alzheimer’s disease. Bioinformatics analysis suggests that polyalanine stretches in the IDRs of RBPs may have evolved to promote RBP assembly.

## 1. Introduction

The human genome encodes more than 1500 RNA-binding proteins (RBPs) [1]. In addition to canonical RNA-binding domains, most, if not all, RBPs have intrinsically disordered regions (IDRs) [2,3,4,5], whose importance has only recently come to light [6,7]. Pioneering studies have shown that some RBPs (including FUS, hnRNPA1, hnRNPA2 and TIA1) use their IDRs to form “dynamic fibers” that may play a role in organizing membraneless cellular structures, such as RNA granules and stress granules [8]. Another function mediated by IDRs is liquid–liquid phase separation (LLPS), which has recently been shown to govern the coordination of cellular membraneless organelles [9,10]. RBPs are the protein family whose IDR-mediated LLPS has been the most studied, notably in terms of the effect of mutations on functional assembly and pathological aggregation [11,12], interactions with other molecules that disrupt or promote self-assembly [13,14], posttranslational modification effects [15,16], residues or sequence motifs that promote condensation [17,18], and their regulation in alternative splicing [19,20]. In spite of the growing literature on these IDRs, residue-specific information, which would provide crucial functional insights, is available for just a few members [21,22,23,24] of this 1500+ family. More structural studies of these IDRs are required to improve our understanding and deduce family-wide rules.

Musashi-1 is a particularly interesting RBP. Its roles in mediating stem cell regeneration and cell differentiation are well-known [25], and recent evidence suggests it may be implicated in neurodegenerative diseases [26,27], as have the prominent RBPs TDP-43 and FUS [28,29]. The removal of Musashi-1′s IDR prevents this protein’s recruitment to stress granules that reduce the chemoresistance of some types of cancer cells [30,31]. This role in granule formation resembles those of other neurodegeneration-associated RBPs; for example, FUS forms liquid-like compartments under stress that recruit proteins to repair damaged DNA, but can also transition to a fibrous, pathological state [11]; likewise, hnRNPA1 acts as a scaffold for stress granules, but this can also lead to pathological aggregation [12].

**Figure 1:**
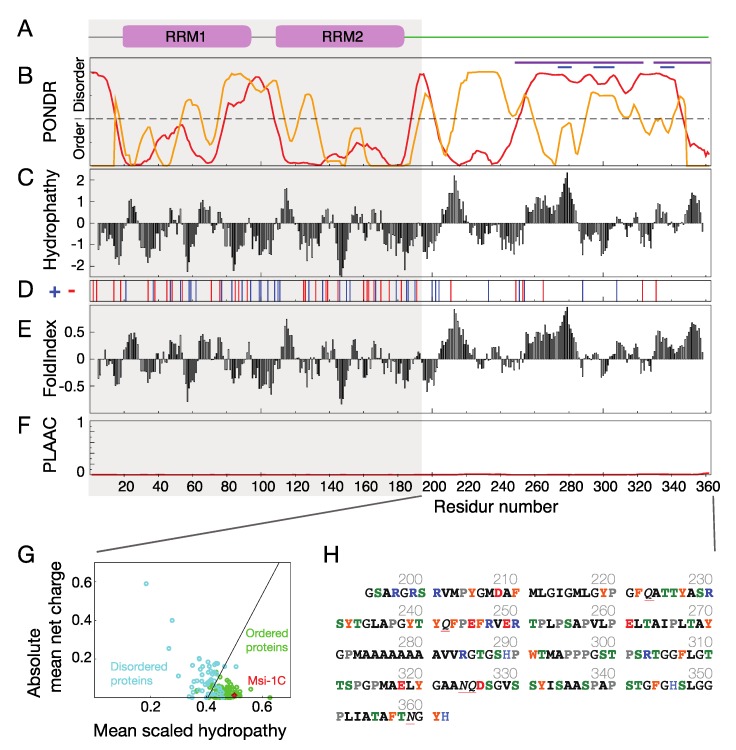
Primary sequence analysis of Musashi-1. (**A**) Functional domains predicted by PROSITE [32] and deposited structures. (**B**) Levels of protein disorder predicted by the PONDR VLXT (red) and XL1_XT (yellow) algorithms [37]. The two regions with low sequence complexity predicted using the SEG algorithm [35] are shown with blue and purple bars. (**C**) Hydropathy according to the Kyte and Doolitte scale (average window size: 9). The scale of each amino acid is derived from experimental observations from solved structures [38]. (**D**) Positions of positively (blue) and negatively (red) charged residues. (**E**) FoldIndex folding [36]. (**F**) Prion-likeness predicted by the PLAAC algorithm [39]. (**G**) Charge–hydropathy plot for a database of known disordered proteins (blue) and folded proteins (green) and the residues 194–362 of Musashi-1 (red) [37]. (**H**) The primary sequence used in this study, with residues labeled according to their properties (blue: positive; red: negative; green: potential phosphorylation sites; orange: aromatic residues; gray: prolines; black: hydrophobic residues).

Musashi-1′s IDR differs in its primary sequence from many of those that have been studied to date. Although its RNA recognition motifs (as predicted by PROSITE [32] and in experimentally solved structures [33,34]) and low sequence complexity (predicted by the SEG algorithm [35]) are similar to those of other known RBPs, the levels of structural disorder predicted for the C-terminal part (residues 194–362; Figure 1A,B) vary depending on the algorithm used. This is probably because of the predominance of hydrophobic residues and the low proportion of charged residues (Figure 1C,D), which are features of folded proteins—as also shown by FoldIndex predictions [36] and its location in the charge-hydropathy plot (Figure 1E,G). Unlike other RBPs implicated in neurodegenerative diseases (see Supplementary Figure S1 for examples), it is not prion-like (Figure 1F). Another unusual feature of its primary sequence is the presence of a polyalanine tract (eight consecutive alanines) in the center, and contrary to the IDRs of many other RBPs [4,5], there are no SR-repeats, RG/RGG motifs, Q/N-rich stretches, or [G/S]Y[G/S] motifs (Figure 1H). How this unique sequence is involved in neurodegenerative disease and/or stress granule recruitment are questions that remain to be answered. In this study, we used NMR spectroscopy to obtain residue-specific information on the IDR, revealing two parts with α-helical propensity. In combination with circular dichroism (CD) and micrography data, the NMR results suggest that the helices are involved in self-association. More generally, the fact that one of the helices in the polyalanine stretch is also found in many other RBPs suggests that polyalanine tracts may have evolved as a means of self-assembly. 

## 2. Results and Discussion

### 2.1. Chemical Shift Assignment of Musashi-1

**Figure 2:**
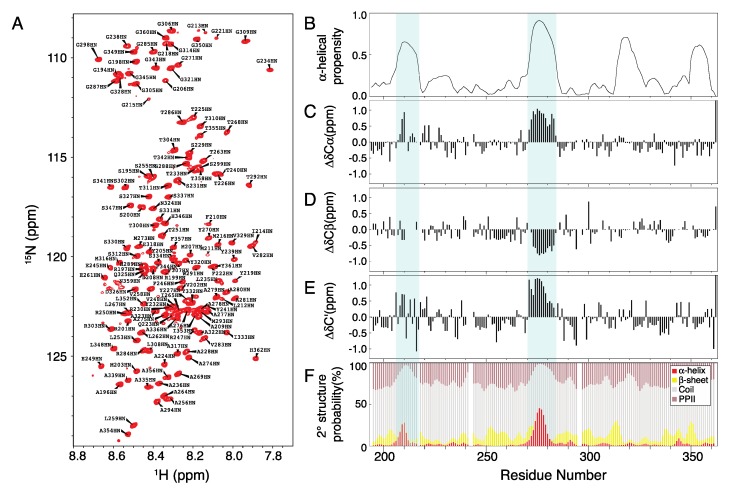
NMR chemical shift assignment of Musashi-1. (**A**) HSQC spectrum of Musashi-1 at 283 K and pH 5.5 with peak assignments. Except L217, Q242, F243, and L319, all non-proline peaks are assigned. (**B**) Residue-specific α-helical propensities predicted using the PASTA program. (**C–E**) Secondary chemical shifts for Cα (**C**), Cβ (**D**), and C’ (**E**) atoms. (**F**) Stacked plot of secondary structure probability based on the assigned chemical shifts, calculated using the δ2D algorithm (red: α-helix; yellow: β-sheet; gray: random coil; brown: polyproline II helix).

The chemical shift assignments for the IDRs of Musashi-1 (residues 194–362; BMRB access number: 50204) are shown in Figure 2A (pH 5.5, 283 K). The protein is prone to aggregation and proline-rich (~12% in the sequence), so a specific assignment strategy was used, as described in the Material and Methods section and the Supplementary Information. Although the PASTA algorithm [40] predicts four α-helices based on the primary sequence (Figure 2B), secondary chemical shift analysis indicates that just two regions, residues ~208–218 and ~270–284, form transient α-helices (Figure 2C–E), with populations (predicted using the δ2D algorithm [41] derived from the molecular dynamics simulation and experimental observations of conformational equilibrium of multiple states, using Cα, Cβ, C’, HN, and NH chemical shifts) of 28% and 45% at most (Figure 2F).

### 2.2. Musashi-1 Self-Association

**Figure 3:**
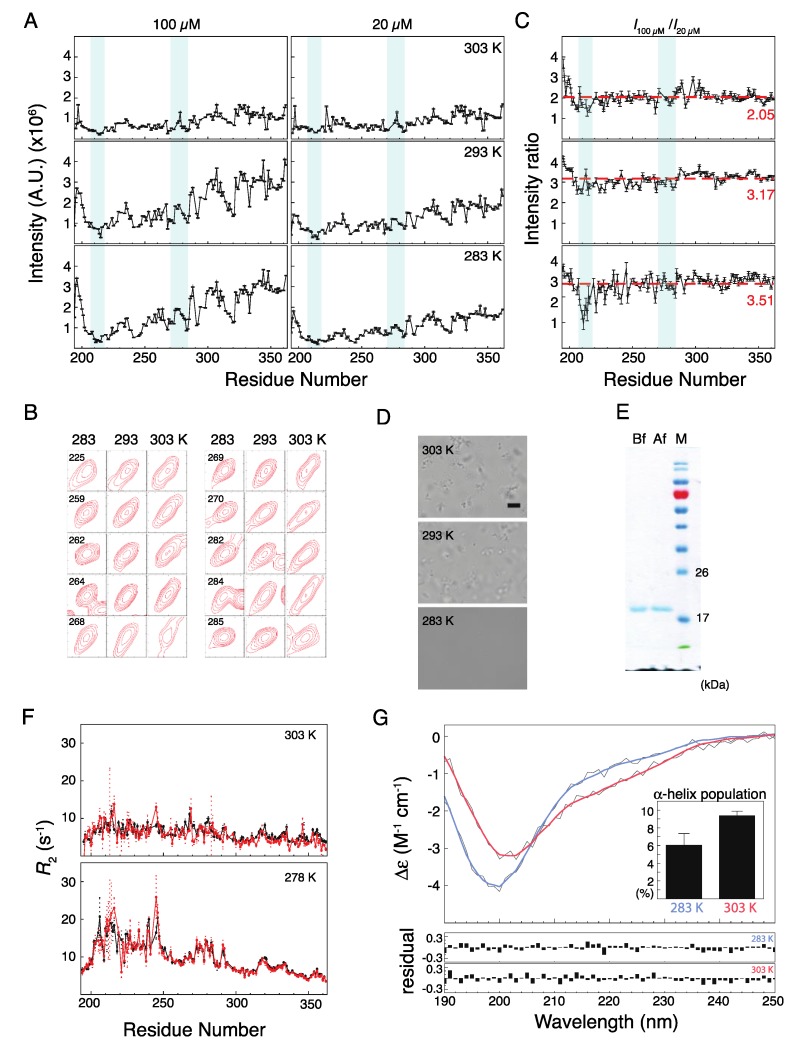
Temperature and concentration dependence of Musashi-1 oligomerization. (**A**) NMR HSQC peak intensities measured at two concentrations (100 and 20 µM) and three temperatures (283, 293, and 303 K) at pH 5.5. Regions with α-helical propensity are shaded blue. (**B**) Selected peaks showing evidence of line-broadening with increasing temperature. The residue number is indicated on the left-upper corner in each panel. (**C**) Peak intensity ratios between 100 and 20 µM samples. The numbers (red) indicate the averaged values. (**D**) Micrographs of the oligomers formed at 293 and 303 K (scale bar = 10 µm) of 20 µM samples. (**E**) SDS-PAGE analysis of protein integrity before (Bf) and after (Af) having increased the temperature above the oligomerization threshold. (**F**) Transverse relaxation rate constants (*R*_2_) at 278 and 303 K for 20 µM (red) and 100 µM (black) samples. (**G**) Circular dichroism spectra for 20 µM samples (pH 5.5) at 283 K (blue) and 303 K (red) fitted using the BeStSel algorithm (gray lines: raw data). The inset compares the average α-helical populations at the two temperatures (three repeats; mean ± SD). Fitting residuals are shown in the lower panel.

The implication of Musashi-1 in neurodegenerative diseases and the role of its IDR in stress granule recruitment [30,31] suggest that it has a propensity to self-assemble as observed in many RBPs [11,12,23]. The HSQC spectra measured for 20 and 100 µM protein samples at pH 5.5, under different temperatures (Figure 3A), show decreased intensities in the N-terminal region, between the two α-helical elements. Many of the peaks in the HSQC spectra have distorted line shapes, notably those from residues in between and within the two helical stretches, because of the kinetics among many conformations (monomer and oligomer) in the intermediate exchange regime (Figure 3B; representative peaks that are not distorted or broadened are shown in Supplementary Figure S2). The peak intensity ratios between the two protein concentrations are less than the corresponding molar ratio (Figure 3C). Furthermore, the average intensity ratio is lower at higher temperatures (3.51, 3.17, and 2.05 at 283, 293, and 303 K, respectively, compared with a molar ratio of 5). At 283K, the intensity ratios in the first α-helical region are even lower, suggesting that the dynamics of this region are reduced at higher concentrations because of stronger self-association. This is not observed at the other two temperatures, because higher temperatures promote the formation of NMR-invisible oligomers at the higher concentration, which reduce the intensity ratio uniformly for all residues, thereby masking the residue-specific soluble monomer/multimer equilibrium effect. Micrographs of the oligomers that form at 293 and 303 K are shown in Figure 3D. The oligomers do not disappear when the temperature is reduced, and as a result, once the samples were heated to 303 K, the NMR intensity remained low when the temperature was decreased back to 283 K (Supplementary Figure S3). SDS-PAGE data confirm that this was not because of protein degradation (Figure 3E). The fact that the transverse relaxation rate constants (*R*_2_) are similar for these two concentrations at all temperatures suggests that the concentration-related decrease in intensity is unlikely due to chemical exchange (e.g., a monomer–oligomer equilibrium; Figure 3F), and confirm that the reduced NMR intensity at higher temperatures is due to the formation of oligomers. Figure 3G shows the CD spectra of 20 µM samples recorded at different temperatures. The α-helical propensities estimated using the BeStSel algorithm [42,43] are 6.0 ± 1.3% at 283 K and 9.4 ± 0.5% at 303 K (Figure 3G inset). This increase in α-helical population may be sufficient to form the oligomers observed micrographically (Figure 3D).

### 2.3. Formation of Musashi-1 Oligomers in a Time-Dependent Manner and at a Higher pH

**Figure 4:**
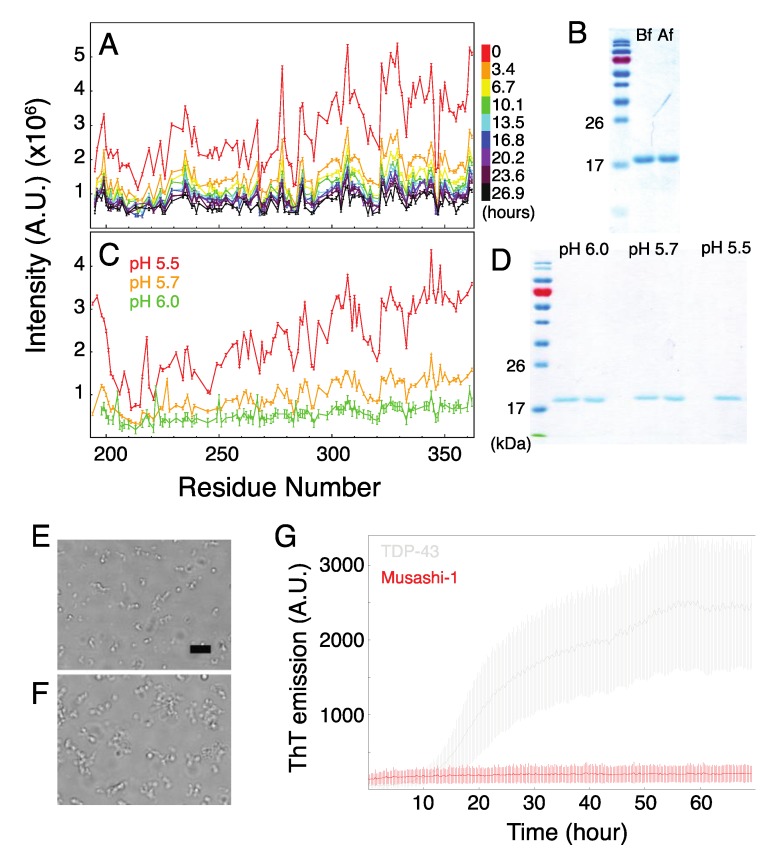
Analysis of the non-cross-beta Musashi-1 oligomers formed at lower pHs and after prolonged incubation. (**A**) Decrease in NMR HSQC peak intensity as a function of time. Data collected at 303 K, pH 5.5. (**B**) SDS-PAGE analysis of protein integrity before (Bf) and after (Af) the one-day NMR experiments, whose results are shown in panel A. (**C**) NMR HSQC peak intensities at 283 K and pH 5.5, 5.7, and 6.0 and 283 K. (**D**) SDS-PAGE analyses of the samples prepared at pH 5.5, 5.7, and 6.0. (**E,F**) Micrographs of samples (**E**) incubated at 303 K for 24 h at pH 5.5 or (**F**) freshly prepared at pH 6. Scale bar = 10 µm. (**G**) ThT assay of Musashi-1 (red) and the C-terminal domain of TDP-43 (control experiment; gray).

Figure 4A illustrates how the NMR peak intensities decreased over time: by more than 80% overall after 24 h for the 100 µM sample. SDS-PAGE shows that this is not because of sample degradation (Figure 4B). The NMR signal intensity also decreased overall on increasing the pH of the sample to 5.7 or 6 (Figure 4C), again, not because of protein degradation (Figure 4D). These results suggest that increasing the pH alters the balance between electrostatic repulsion (from the net positive charge (+2) of the IDR) and hydrophobic attraction in favor of the latter, leading to oligomerization. Similar assembly behavior was observed by using microscopy after longer incubation times at pH 5.5 or a freshly prepared sample at pH 6 (Figure 4E,F). No fluorescence signals were observed in ThT assays, indicating that there are no cross-beta structures in the oligomers (Figure 4G). (Note that fluorescence signals from cross-beta structures were observed in control ThT assays performed on the C-terminal ID domain of TDP-43). The precipitation of the protein made it difficult to use CD spectroscopy to estimate its secondary structure, as the Δε values (Equation (1)) used as input for the program became more uncertain as aggregates formed and the signal intensity decreased. 

### 2.4. The Role of Polyalanine Tracts in Musashi-1 and in RBPs in General

The structured RNA recognition domains of RBPs have binding pockets that have evolved to identify specific nucleic acids sequences. However, how RBPs are recruited to the gene-regulation machinery remains largely unknown. Most RBPs have IDRs whose role in controlling the entire proteins’ location has only recently come to light. The features of these IDRs, including [G/S]-[F/Y/W]-[G/S] motifs [17,44], cation-π interactions [16,18], charge–charge interactions [45], and prion-like behavior [46], lead them to assemble in response to environmental changes. Polyalanine tracts may play a similar role in the regulation of RBP assembly, as shown here for Musashi-1. Polyalanine tracts have been implicated in many diseases [47] and can act as nuclear export signals [48]. The assembly of polyalanine stretches into α-helical clusters without forming amyloid-fibrils has also been demonstrated in both model peptides and proteins with polyalanine tracts [49]. Recent studies on aggregation-prone proteins and those with prion-like domains have shown that forming higher-order oligomers is their physiological role, albeit at the price of pathological aggregation, when the process is perturbed [50]. The same reasoning can be applied to polyalanine tracts, in the sense that, although they can induce the formation of oligomers that in turn promote amyloid formation [49], they may also have physiologically important roles in the formation of higher-order assemblies.

**Figure 5:**
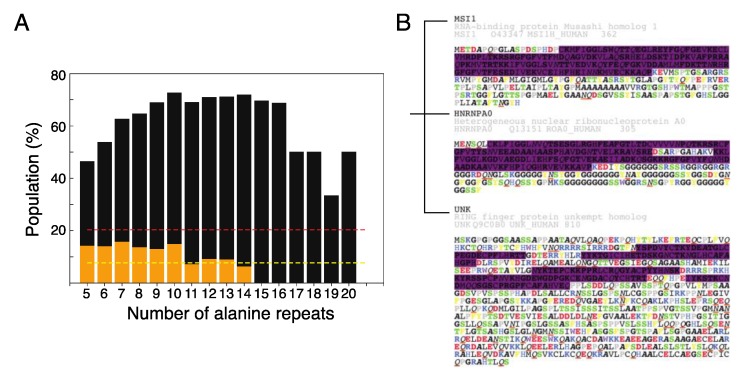
Polyalanine tracts are a common feature of RNA related proteins, and may be one of the ways in which RBPs self-assemble through their IDRs. (**A**) Bioinformatics analysis of the proportions of human proteins with different lengths of alanine repeats (from 5 to 20) either related to RNA function (black bars; the red dashed line shows the proportion overall) or specifically labeled “RNA binding” (orange bars; overall proportion, yellow dashed line). (**B**) Illustration of the differences in the IDRs of RBPs that bind to similar RNA motifs. The purple shaded regions are those identified by PROSITE as RNA recognition motifs. The amino acids are colored according to their physical properties (positive charge: blue; negative charge: red; F/Y: yellow; W: purple; S/T (potential phosphorylation site for the addition of negative charges): green; P: gray; A: italic-bold black; Q/N: red underlined italic).

Among the 20,000+ human protein sequences in the UniProtKB/Swiss-Prot database, gene ontology (GO) annotations indicate that about 20.3% (listed in the Supporting Information) are RNA-related (including RNA polymerases associated functions), and 7.7% are specifically related to “RNA binding”. The proportion of proteins with polyalanine tracts (5–20 repeats) that are RNA-related is much higher; for example, more than 60% of those with 7–16 repeats are associated to RNA-related function. Only considering proteins tagged as “RNA binding”, the proportion of proteins with stretches of 5–10 alanine repeats can be twice as high as in the database as a whole (Figure 5A; Supplementary Table S2). There is, therefore, an association between the presence of polyalanine tracts and RNA-related function.

In a recent study [51], Dominguez et al. purified the RNA recognition domains of a representative group of RBPs, identified their interacting RNA motifs, and then hierarchically clustered the RBPs in terms of their RNA-binding motifs. Roughly a quarter of these RBPs (18 out of 76) have polyalanine tracts ranging from 5 to 13 repeats (Supplementary Table S3), in agreement with our hypothesis that polyalanine tracts are one of the many features of RBP IDRs. More importantly, while those RBPs bind to similar RNA motifs, the physicochemical properties of their disordered regions have little in common. For example, the polyalanine feature of Musashi-1 (see Reference [51]) is not found in the IDRs from the same groups of RBPs that binds similar RNA-motif: UNK (which has charged blocks) or hnRNPA0 (which has G/S-F/Y/W-G/S motifs; Figure 5B), and there are other examples in other RNA-motif groups (Supplementary Figure S4). Even though some RBPs have similar RNA-binding motifs, their IDRs may have evolved different physical properties that ensure specific spatiotemporal regulation. 

This focus on continuous polyalanine tracts may be too restrictive, and the above analysis may have underestimated the importance of α-helix. Many other types of amino acids can form α-helices, and many polyalanine tracts in the database are non-continuous. For example, the IDR of TDP-43 has a transient α-helical region [23] (primary sequence, AMMAAAQAA) that is involved in self-association. Furthermore, a disrupted polyalanine tract may still contribute to self-assembly in combination with a few [G/S]-[F/Y/W]-[G/S] motifs or an increased prion-like propensity (Supplementary Figure S1). 

## 3. Material and Methods

### 3.1. Musashi-1 Primary Sequence Analysis

The primary sequence of full-length Musashi-1 was taken from the UniProt database (entry number O43347) and used as input for the programs PROSITE [32], PONDR [37], ProtScale [52], FoldIndex [36], PLAAC [39], and PASTA [40], to predict the properties of the protein. 

### 3.2. Polyalanine Tracts Analysis

The UniProt Knowledgebase of manually curated human protein information (UniProtKB/Swiss-Prot, entries for 20367 human proteins as of 8 January 2020) was downloaded from the UniProt website [53]. Proteins with different lengths of polyalanine tracts were extracted, using the “Peptide search” function on the same website. Proteins with RNA-related keywords in gene ontology annotations were counted, using an in-house script. 

### 3.3. DNA Construct

The cDNA of Musashi-1 was provided by Dr. Wei-Yi Chen (NYMU). The C-terminal domain (residue numbers 194 to 362) was cloned to a pET-21 vector, using *NdeI* and *XhoI* restriction enzymes. A hexa-histidine tag was added to the N-terminus for purification. No further non-native residues were added. To assist NMR assignment, four constructs with deleted fragments were prepared, using the primers listed in Supplementary Table S1. All constructs were fully sequenced. 

### 3.4. Protein Expression and Purification

Single colonies of transformed *E. coli.* BL21 (DE3) or Rosetta were selected from an ampicillin agar plate and used to inoculate 5.0 mL of lysogeny broth containing 0.1 mg·mL^−1^ ampicillin. The pre-culture was left in a 37 °C shaker, overnight, and used to inoculate 500 mL of lysogeny broth or minimal M9 medium (containing ^15^NH_4_Cl and/or ^13^C-glucose for isotope labeling). The growth cultures were left in a 37 °C shaker and shaken vigorously until the O.D_600_ reached 0.6. The cells were induced with a final concentration of 1 mM isopropyl β-D-1-thiogalactopyranoside and left shaking at 25 °C, overnight. The harvested cells were lysed by sonication in 20 mM Tris buffer at pH 8. After centrifugation (Beckman JA25.5, 30 min, 18,000 rpm, at 4 °C), the inclusion bodies were dissolved with the same lysis buffer, with an additional 8 M urea. After the second centrifugation (10,000 rpm, 30 min), the supernatant was passed through a 0.45 µm filter and loaded onto a nickel-charged immobilized metal-ion affinity chromatography column (Qiagen, Hilden, Germany). The column was washed with ten volumes of 20 mM Tris buffer with 8 M urea at pH 8 to remove non-specific binding proteins. The target protein was eluted with an additional 500 mM imidazole in the same wash buffer. The eluted sample was acidified, using trifluoroacetic acid, to a pH lower than 3, and then loaded into a C4 reverse-phase column (Thermo Scientific Inc., MA, USA). The protein was eluted with an increasing gradient of acetonitrile mixed with triple-distilled water, using an HPLC system. The eluted sample was immediately frozen with liquid nitrogen and lyophilized. The lyophilized sample was stored in a drying cabinet, until use.

### 3.5. Chemical Shift Assignment

The assignment experiments were performed on a Bruker AVANCE 800 MHz spectrometer, at 283 K. A denaturation-then-titration strategy (assigning the protein under harsh conditions and then titrating to physiological conditions; see Supplementary Information) was used to assign the chemical shifts of the protein. In addition to standard pulse sequences (HNCO, HN(CA)CO, HNCA, HN(CO)CA, HNCACB, and CBCA(CO)NH), we also used (H)N(COCO)NH and (HN)CO(CO)NH [54] to complete the sequential assignments of long-range (*i*, *i*+2) backbone nitrogen and carbonyl carbon atoms separated by prolines. Different fragments were created to confirm the assignment (see Supplementary Information). Secondary chemical shifts were analyzed by using Kjaergaard et al.’s database of random coil shifts [55]. Secondary structure probabilities based on the chemical shifts were calculated by using the δ2D program [41]. 

### 3.6. NMR Experiments and Analysis

The urea and pH titration experiments were monitored, using HSQC spectra [56] (with WATERGATE [57] solvent suppression) recorded on Bruker AVANCE III 600 and 850 MHz spectrometers. All the samples were freshly prepared: The estimated amount of lyophilized sample required was dissolved in 20 mM MES buffer with protease inhibitor cocktail (Roche, Basel,, Switzerland); the samples were centrifuged at 12,000× *g* for 5 min, to remove any aggregates; and the concentration was confirmed by measuring the absorbance at 280 nm on a NanoDrop spectrometer (Thermo Scientific Inc., MA, USA). Transverse relaxation rate constants were measured on the 850 MHz spectrometer, using standard pulse sequences [58] (with delays of 17.2, 34.3, 51.5, 68.6, 120.1, and 154.4 ms). Peak intensities were fitted to exponential decays with a Monte Carlo procedure, to estimate fitting error. All dynamics data were collected in an interleaved manner, with an inter scan delay of 3 s. 

### 3.7. Circular Dichroism Spectroscopy

Circular dichroism spectra were recorded by using an AVIV model 410 spectropolarimeter. A 0.1 mm cuvette was used. Data were collected between 190 and 260 nm, with an interval of 1 nm. Ten measurements were co-added for each data point, and all the experiments were performed in triplicate. The measured theta machine units (*θ*) were converted to Δ*ε*, using the following equation [59]:

(1) $$\Delta \varepsilon =\theta \times \frac{0.1\times MRW}{l\times C\times 3298}$$

where MRW is the mean residue weight (molecular weight/residue number, in Dalton), *l* is the path length (in cm), and *C* is the protein concentration (in mg/mL). The resulting curves were fitted, using the program BeStSel [42,43], to estimate secondary structure populations. Samples (20 µM) were prepared in 20 mM MES buffer at pH 5.5, at different temperatures, otherwise indicated.

### 3.8. Microscopy 

Micrographs were collected by using an Olympus BX51 device with a 40× long working-distance objective lens, and the images were recorded with a Zeiss AxioCam MRm camera. The samples were placed on a thermostatic microscope stage (THM120, Linkam Scientific Inc., Tadworth, U.K.) and were equilibrated for 5 min, before collecting the images.

### 3.9. Thioflavin T Fluorescence Assay

A 1 M stock solution of Thioflavin T (ThT, Acros organics) was prepared in 90% (*v*/*v*) ethanol and protected from light prior to use. The ThT stock was diluted, using 20 mM MES-NaOH buffer to obtain a 1 mM ThT working solution. Protein sample solutions were mixed with the ThT working solution to the designed final concentration. Each sample (120 µL) was distributed in a black 96-well plate. The plates were sealed and transferred to an Infinite 200 (TECAN) multimode microplate reader and incubated at 30 °C. The emission intensity at 480 nm was recorded, using slit widths of 5 nm for both excitation and emission. At least three independent experiments were performed for each condition. 

## 4. Conclusions

While developments in cryogenic electron microscopy [60,61,62,63] have revolutionized structural biology [64], NMR spectroscopy remains the most powerful tools to study protein dynamics, especially IDRs and IDPs, with residue-specific resolution [65]. RNA-binding proteins are a large family of proteins with high sequence variability in their IDRs. This study of Musashi-1 illustrates the power of NMR spectroscopy to study disordered proteins. The assignment strategy we used can be applied to many other aggregation-prone proteins, and the chemical shift assignments here can be used to conduct further studies of how its IDRs interact with binding partners [66,67]. Indeed, some identified interacting regions cover the first α-helix, which might be critical for their interaction [66,67]. Our results highlight Musashi-1′s ability to self-assemble through interactions involving the region between the two α-helical elements. They also provide a mechanistic explanation of how this protein forms pathological oligomers and is recruited into stress granules via its polyalanine tracts. Polyalanine tracts are found in the IDRs of many other RBPs, suggesting that they have evolved as a means to control where and when RBPs self-assemble. 

## Accession Numbers

The backbone chemical shift assignments of Musashi-1 were deposited in the BMRB, under accession code 50204.

## Supplementary Materials

Supplementary materials can be found at https://www.mdpi.com/1422-0067/21/7/2289/s1.

## Abbreviations

RBP	RNA-binding proteins
IDP	Intrinsically disordered proteins
NMR	Nuclear magnetic resonance
CD	Circular dichroism
HSQC	Heteronuclear single-quantum coherence
SDS-PAGE	Sodium dodecyl sulfate–polyacrylamide gel electrophoresis
