# Supplementary material for: Musashi-1: An Example of How Polyalanine Tracts Contribute to Self-Association in the Intrinsically Disordered Regions of RNA-Binding Proteins
Source: Int J Mol Sci. 2020 Mar 26;21(7):2289. doi: 10.3390/ijms21072289 (PMC7177541; doi:10.3390/ijms21072289)
Supplement: Supplementary file 1 [file ijms-21-02289-s001.pdf]

Supporting Information

Musashi-1: an example of how polyalanine tracts contribute to self-association in the intrinsically disordered regions of RNA-binding proteins

Tsai-Chen Chen and Jie-rong Huang

Institute of Biochemistry and Molecular Biology and Institute of Biomedical Informatics,

National Yang-Ming University, No. 155 Section 2 Li-nong Street, Taipei, Taiwan

Assignment strategy

We followed a denaturation-then-titration strategy – assigning the protein under harsh conditions and then titrating back to physiological conditions – because Musashi-1's IDR has a strong tendency to aggregate. We also used (H)N(COCO)NH and (HN)CO(CO)NH pulse sequences to help complete the sequential assignment, using long-range (*i*, *i*+2) connections between backbone nitrogen and carbonyl-carbon atoms to overcome disruptions due to the 20 prolines (which make up about 12 % in the primary sequence).

We first prepared the 0.7 mM $^{15}N/^{13}C$-labeled sample in 10 mM glycine buffer with 8 M urea at pH 2.5, conditions under which the NMR peaks are well-dispersed (Figure A1). The assignment was facilitated by (H)N(COCO)NH and (HN)CO(CO)NH data. ^{15}N-labeled samples of four different constructs were used to distinguish assignments that remained ambiguous (Figure A2) under the same buffer condition. ^{15}N-labeled sample (~70 μM) in 10 mM phosphate buffer at pH 5.5 was titrated to different pHs till pH=2.5 using phosphoric acid (Figure A3). ^{15}N-labeled samples (~70 μM) in 20 mM MES buffer at pH 5.5 with urea concentrations ranging from 0 to 8 M are shown in Figure A4. Finally, all the triple resonance assignment experiments were applied to the $^{15}N/^{13}C$-labeled sample (~130 μM) in 20 mM MES at pH 5.5 to confirm the assignment.

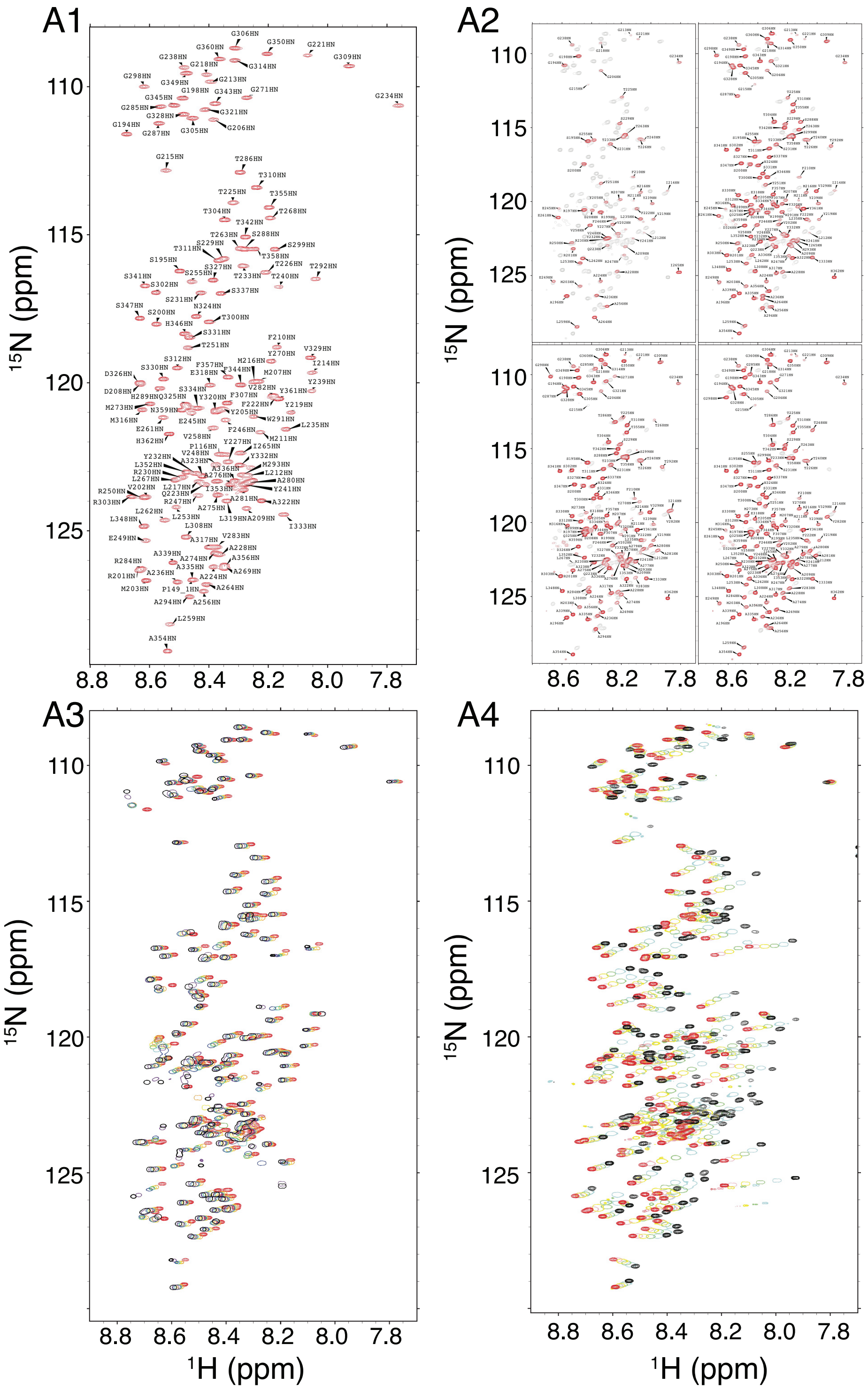

A1. Chemical shift Assignment in the presence 8 M urea at pH 2.5. **A2**. HSQC spectra of four different truncated contracts (red, also see Table S1) overlaid with the wild-type (grey). **A3.** Titration of pH from 2.5 (red) to 5.5 (black) in the presence of 8 M urea. **A4**. Titration of urea from 8 M (red) to zero (black) at pH 5.5.

Supporting Figures:

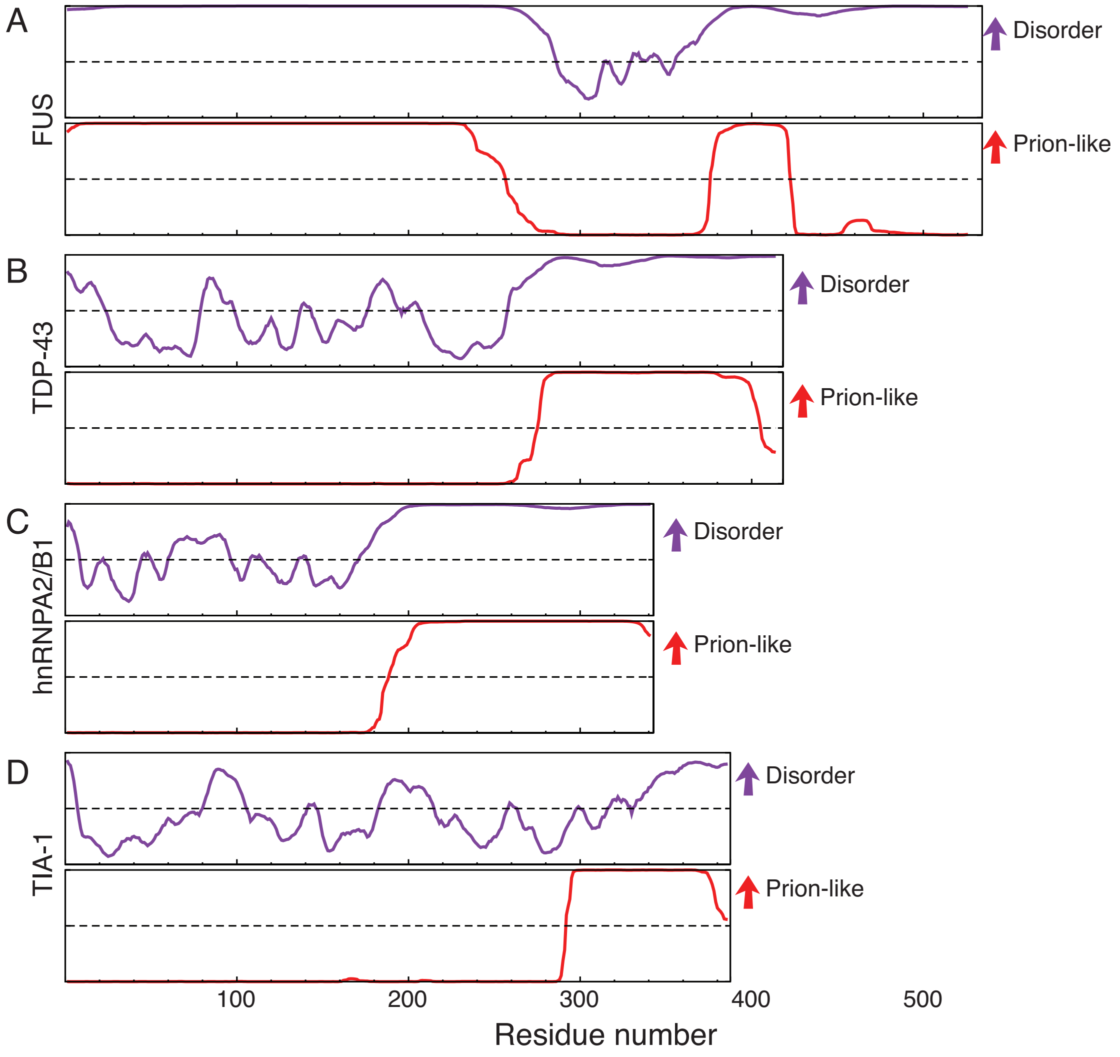

Figure S1. RNA binding proteins involved in neurodegenerative diseases have intrinsically disordered regions (purple) with prion-likeness (red). (**A**) FUS, (**B**) TDP-43, (**C**) hnRNPA2/B1, (**D**) TIA-1.

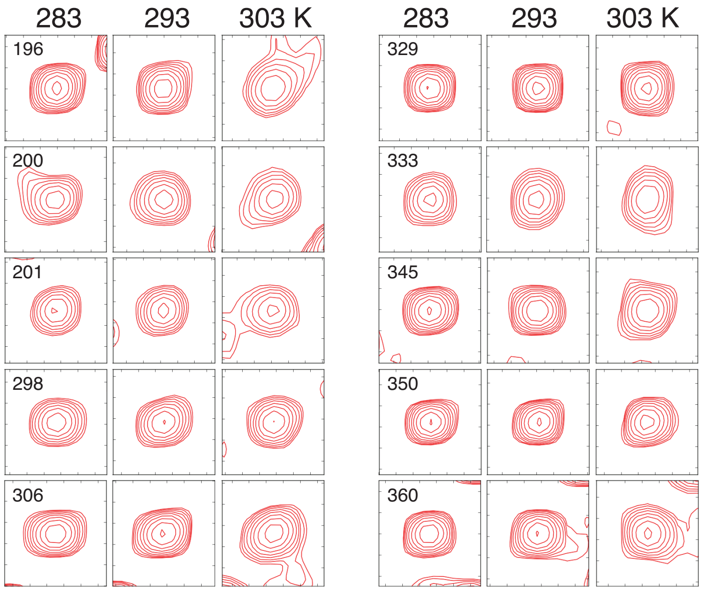

Figure S2. In contrast with Figure 3B: examples of peaks in the HSQC spectra that show little line-broadening or distortion at any of the three temperatures.

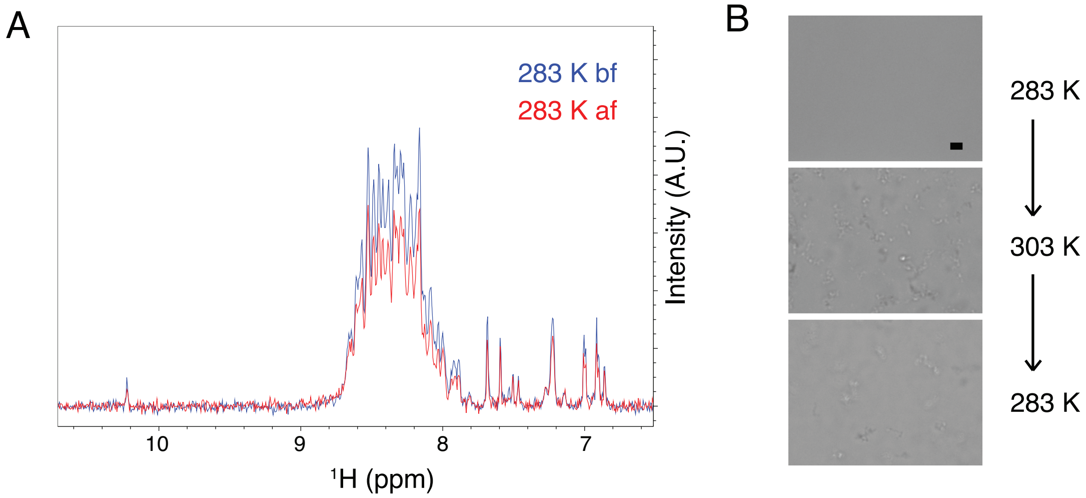

Figure S3. Irreversibility of Musashi-1 oligomerization. (**A**) NMR spectra of Musashi-1 samples before (blue) and after heating to 303 K (red). (**B**) Sequence of micrographs of a (fresh) Musashi-1 sample at 283 K, 303 K and then 283 K once again. Scale bar: 5 μm.

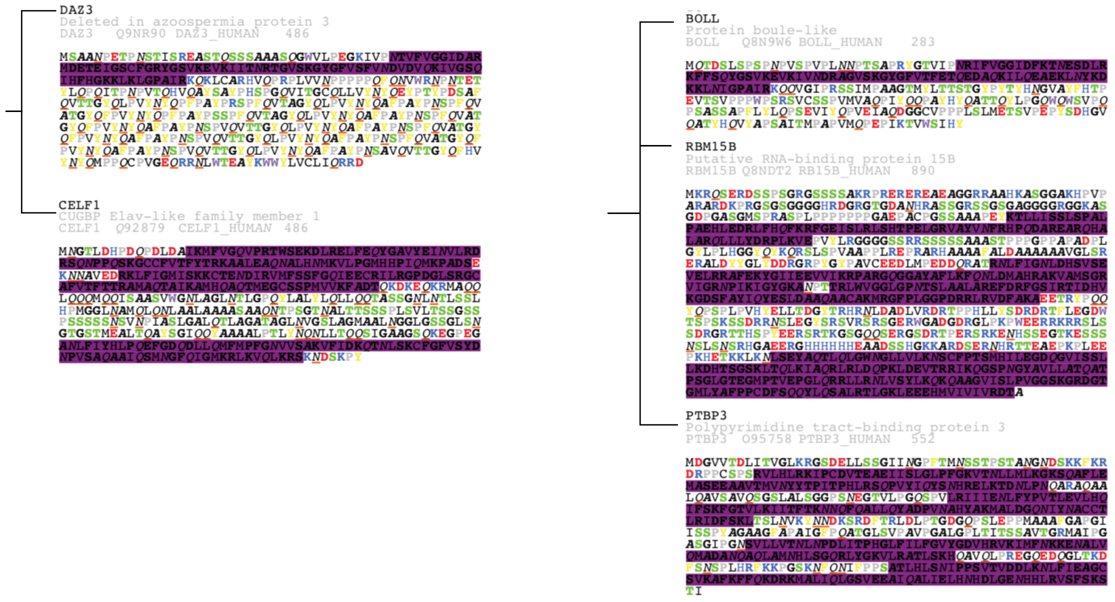

Figure S4. Examples of RBPs that bind to similar RNA motifs but whose IDRs have different properties. The amino acids are coloured according to their physical properties (Positive charge: blue; negative charge: red; F/Y: yellow; W: purple; S/T (potential phosphorylation site for the addition of negative charges): green; P: grey; A: italic-bold black; Q/N: red underlined italic)

Supporting Information: Tables

Table S1. Primers used in this study.

Constructs	Primers
His-MSI-1C<br>a.a. 194-362	Fw-5'-AAAAAACATATGCATCATCATCATCATCATGGCAGCGCGCGC-3' (*NdeI*)<br>Rv-5'-AAAAACTCGAGTCAGTGGTACCCA-3' (*XhoI*)
His-MSI-1CΔS1<br>247-265 deleted	Fw-5'-CCCGAATTCCCTCTCACTGCCTACGGACCA-3'<br>Rv-5'-AGTGAGAGGGAATTCGGGGAACTGGTAGGT-3'
His-MSI-1CΔS2<br>287-297 deleted	Fw-5'-GGGACAGGTTCGACTCCCAGCCGCAC-3'<br>Rv-5'-CGAACCTGTCCCTCGAACCACAGCCG-3'
His-MSI-1CΔA2<br>266-286 deleted	Fw-5'-ACAGCCATTGGCTCTCACCCCTGGACGATG-3'<br>Rv-5'-GTGAGAGCCAATGGCTGTAAGCTCGGGGAG-3'
His-MSI-1Cnt<br>a.a. 194-265	Fw-5'-CATTTAATAAACTGCCTACGGACCAATGGC-3'<br>Rv-5'-AGTTTATTAAATGGCTGTAAGCTCGGGGAG-3'

Table S2. Number of human proteins with different lengths of alanine repeats related to RNA functions.

N-alanine repeats	Number of *N*-alanine-repeats with an RNA-related GO	Number of *N*-alanine-repeats with an "RNA-binding" GO	Total number of proteins containing *N*-alanine repeats
5	319	97	687
6	221	57	411
7	164	41	262
8	115	24	178
9	91	17	132
10	69	14	95
11	49	5	71
12	39	5	55
13	32	4	45
14	23	2	32
15	16	0	23
16	11	0	16
17	3	0	6
18	2	0	4
19	1	0	3
20	1	0	2
21	0	0	1

GO, gene annotation.

Table S3. List of RBPs in Dominguez et al.'s study with polyalanine tracts.

Gene name	Protein name	Entry	*N*-alanine
RBM47	RNA-binding protein 47	A0AV96	14, 6
RBM24	RNA-binding protein 24	Q9BX46	11, 13
RBM4	RNA-binding protein 4	Q9BWF3	10, 5
RBM23	Probable RNA-binding protein 23	Q86U06	9
RBFOX2	RNA binding protein fox-1 homolog 2	O43251	8
MSI1	RNA-binding protein Musashi homolog 1	O43347	8
MBNL1	Muscleblind-like protein 1	MBNL1	7
PUM1	Pumilio homolog 1	Q14671	7, 5
RBM15B	Putative RNA-binding protein 15B	Q8NDT2	6
RBM4B	RNA-binding protein 4B	Q9BQ04	6, 5
RBFOX3	RNA binding protein fox-1 homolog 3	A6NFN3	6
HNRNPDL	Heterogeneous nuclear ribonucleoprotein D-like	O14979	6
KHSRP	Far upstream element-binding protein 2	Q92945	6
SF1	Splicing factor 1	Q15637	5
A1CF	APOBEC1 complementation factor	Q9NQ94	5
HNRNPD	Heterogeneous nuclear ribonucleoprotein D0	Q14103	5, 5
NOVA1	RNA-binding protein Nova-1	P51513	5, 5, 5
PUF60	Poly(U)-binding-splicing factor	Q9UHX1	5, 5

Appendix

Chemical shift assignment

RES		CA	CB	CO	N	HN
194	G	45.006	0.0	173.819	110.654	8.479
195	S	58.085	64.004	174.313	115.761	8.318
196	A	52.533	19.042	177.669	126.252	8.457
197	R	56.145	30.696	176.789	120.460	8.338
198	G	45.049	0.0	173.838	110.044	8.376
199	R	55.938	30.799	176.342	120.578	8.235
200	S	58.231	63.874	174.217	117.318	8.340
201	R	56.119	30.758	175.833	123.025	8.413
202	V	62.279	32.520	175.723	121.367	8.149
203	M	52.736	32.374	174.087	125.521	8.364
204	P	63.067	31.881	176.092	0.0	0.0
205	Y	58.018	38.570	176.271	119.824	8.181
206	G	45.149	0.0	174.316	110.905	8.230
207	M	55.819	32.523	176.096	119.738	8.108
208	D	54.819	40.898	176.431	120.403	8.291
209	A	53.525	18.616	178.275	122.840	8.062
210	F	0.0	39.047	176.473	118.164	8.014
211	M	0.0	0.0	175.085	119.721	8.017
212	L	55.217	0.0	177.810	121.875	7.945
213	G	45.380	0.0	174.205	108.570	8.031
214	I	61.645	0.0	176.803	119.044	7.773
215	G	45.422	0.0	174.184	111.882	8.310
216	M	55.325	32.852	175.397	119.205	7.969
217	L	0.0	0.0	177.508	0.0	0.0
218	G	44.840	0.0	173.258	109.036	8.219
219	Y	55.881	38.008	174.144	121.015	7.878
220	P	63.774	31.675	177.166	0.0 0.0	
221	G	45.160	0.0	173.895	108.794	7.975
222	F	58.275	39.489	175.524	120.327	7.999
223	Q	55.425	29.498	174.981	122.706	8.189
224	A	52.758	19.150	177.961	125.211	8.231
225	T	62.023	69.716	174.723	112.755	8.083
226	T	61.908	69.725	174.281	115.626	7.964
227	Y	58.229	38.581	175.716	122.010	8.114
228	A	52.635	19.112	177.545	124.590	8.119
229	S	58.551	63.656	174.636	114.563	8.104
230	R	56.164	30.557	175.981	122.332	8.202
231	S	58.124	63.766	173.963	115.968	8.165
232	Y	57.909	38.758	175.900	122.287	8.189
233	T	61.763	69.693	174.695	115.400	8.077
234	G	45.090	0.0	173.450	110.435	7.715
235	L	54.534	42.450	176.726	121.039	7.957
236	A	50.434	17.997	175.186	126.176	8.251
237	P	63.439	31.831	177.419	0.0 0.0	
238	G	45.125	0.0	173.837	109.209	8.414
239	Y	58.130	39.073	175.663	119.925	7.881
240	T	61.461	70.096	173.549	115.668	7.943
241	Y	58.059	0.0	0.0	122.313	8.010
242	Q	0.0	0.0	0.0	0.0	0.0
243	F	0.0	0.0	0.0	0.0	0.0
244	P	63.337	31.864	176.530	0.0	0.0
245	E	56.624	30.040	175.992	120.300	8.501
246	F	57.468	39.492	175.093	121.040	8.138

247	R	55.595	31.045	175.389	123.456	8.083
248	V	62.234	32.604	175.926	122.243	8.175
249	E	56.182	30.211	176.039	125.257	8.544
250	R	55.762	30.730	176.007	122.624	8.423
251	T	59.916	69.638	172.539	118.662	8.232
252	P	62.887	32.054	176.537	0.0 0.0	
253	L	53.009	41.444	175.437	123.993	8.357
254	P	63.119	31.897	176.731	0.0 0.0	
255	S	58.032	63.747	173.660	115.759	8.277
256	A	50.432	18.199	175.186	126.933	8.196
257	P	62.838	31.954	176.458	0.0 0.0	
258	V	62.082	32.608	175.976	121.356	8.286
259	L	52.708	41.435	175.034	128.212	8.372
260	P	62.988	31.919	176.730	0.0 0.0	
261	E	56.429	30.024	176.532	120.806	8.530
262	L	55.173	42.042	177.387	123.570	8.336
263	T	61.788	69.707	173.860	114.897	8.028
264	A	52.106	19.182	177.160	126.731	8.224
265	I	58.669	38.500	174.518	122.479	8.140
266	P	62.927	32.077	176.777	0.0 0.0	
267	L	55.570	42.019	177.792	122.591	8.401
268	T	61.694	69.707	174.081	113.468	7.912
269	A	52.348	19.192	176.955	125.635	8.154
270	Y	57.803	38.996	176.040	118.801	7.999
271	G	45.018	0.0	172.363	110.154	8.155
272	P	63.967	31.912	178.068	0.0 0.0	
273	M	56.370	32.202	177.006	119.278	8.354
274	A	53.373	18.552	178.697	124.858	8.107
275	A	53.537	18.455	178.961	122.700	8.222
276	A	53.474	18.382	178.963	122.463	8.114
277	A	53.401	18.442	178.887	122.543	8.015
278	A	53.383	18.419	178.725	122.360	8.050
279	A	53.244	18.469	178.510	121.844	7.971
280	A	52.904	18.647	178.038	121.585	7.921
281	A	52.874	18.723	178.213	121.877	7.881
282	V	63.120	32.328	176.652	119.202	7.798
283	V	62.902	32.287	176.376	123.871	8.034
284	R	56.380	30.725	176.786	124.522	8.336
285	G	45.162	0.0	174.357	109.550	8.289
286	T	61.911	69.797	175.267	113.055	8.139
287	G	45.175	0.0	173.942	110.983	8.473
288	S	58.145	63.687	173.831	115.145	8.124
289	H	53.103	28.591	172.430	120.143	8.339
290	P	63.457	31.825	176.508	0.0 0.0	
291	W	57.379	29.152	176.153	120.363	8.065
292	T	61.536	69.916	173.565	116.164	7.816
293	M	55.077	32.947	175.162	122.479	8.052
294	A	50.331	17.906	174.694	127.062	8.262
295	P	0.0	0.0	0.0	0.0	0.0
296	P	0.0	0.0	0.0	0.0	0.0
297	P	63.344	31.832	177.549	0.0	0.0
298	G	45.123	0.0	174.181	109.892	8.557
299	S	58.371	63.988	174.183	115.337	8.063
300	T	59.827	69.718	172.715	118.193	8.272
301	P	63.248	32.049	176.811	0.0	0.0
302	S	58.229	63.715	174.745	116.295	8.419
303	R	56.137	30.696	176.478	123.419	8.492
304	T	61.802	69.675	175.013	114.431	8.177

305	G	45.262	0.0	174.355	111.130	8.370
306	G	44.910	0.0	173.692	108.475	8.195
307	F	57.823	39.492	175.828	120.161	8.159
308	L	55.006	42.236	177.368	124.532	8.304
309	G	45.214	0.0	174.007	109.020	7.834
310	T	61.595	69.848	174.687	113.250	8.049
311	T	61.650	69.818	174.168	116.196	8.209
312	S	56.299	63.302	172.574	119.742	8.360
313	P	63.349	32.097	176.956	0.0	0.0
314	G	44.454	0.0	0.0	109.122	8.196
315	P	63.309	31.950	177.362	0.0	0.0
316	M	55.527	32.455	176.152	120.063	8.451
317	A	52.879	19.105	177.875	124.642	8.160
318	E	56.579	29.888	176.381	119.496	8.302
319	L	55.254	42.245	177.148	0.0	0.0
320	Y	57.902	38.472	176.398	119.965	8.116
321	G	45.198	0.0	173.763	110.309	8.198
322	A	52.469	19.234	177.591	123.642	8.054
323	A	52.509	18.984	177.533	122.508	8.266
324	N	53.243	38.671	175.242	117.368	8.286
325	Q	55.951	29.294	175.667	120.497	8.327
326	D	54.354	41.076	176.365	121.438	8.377
327	S	58.771	63.684	175.147	116.771	8.307
328	G	45.320	0.0	174.173	110.761	8.455
329	V	62.319	32.570	176.374	119.095	7.894
330	S	58.393	63.703	174.534	119.359	8.414
331	S	58.389	63.703	173.913	117.897	8.250
332	Y	58.037	38.582	175.403	122.106	8.094
333	I	60.730	38.639	175.723	123.516	7.918
334	S	58.120	63.793	174.243	120.076	8.260
335	A	52.388	19.146	177.162	126.340	8.307
336	A	52.162	19.185	177.469	122.880	8.166
337	S	56.329	63.159	172.284	116.767	8.204
338	P	62.853	31.953	176.220	0.0	0.0
339	A	50.384	17.829	175.527	125.991	8.404
340	P	62.978	31.979	177.010	0.0	0.0
341	S	58.285	63.625	174.890	116.281	8.492
342	T	61.728	69.741	174.936	114.812	8.105
343	G	45.021	0.0	173.769	110.337	8.273
344	F	57.969	39.410	176.432	120.022	8.179
345	G	45.182	0.0	173.803	110.617	8.407
346	H	55.094	29.181	174.395	118.078	8.239
347	S	58.252	63.726	174.510	117.226	8.403
348	L	55.287	42.105	177.763	124.445	8.479
349	G	45.012	0.0	174.081	109.553	8.382
350	G	44.382	0.0	0.0	108.896	8.069
351	P	62.929	32.104	176.764	0.0	0.0
352	L	55.155	41.983	177.175	122.192	8.332
353	I	60.526	38.452	175.715	122.607	8.060
354	A	52.485	19.180	177.664	128.692	8.407
355	T	61.667	69.833	174.109	113.656	8.044
356	A	52.435	19.073	177.283	125.854	8.206
357	F	57.620	39.310	176.119	119.311	8.181
358	T	61.707	69.716	174.084	115.420	8.049
359	N	53.328	38.642	175.381	120.646	8.335
360	G	45.079	0.0	173.468	108.830	8.225
361	Y	57.961	38.555	174.837	120.314	7.983
362	H	56.718	29.873	178.140	124.925	7.776

Gene ontology annotations related to RNA

GO:0000049; F:tRNA binding; TAS:BHF-UCL.
GO:0000120; C:RNA polymerase I transcription factor complex; TAS:ProtInc.
GO:0000122; P:negative regulation of transcription by RNA polymerase II; TAS:UniProtKB.
GO:0000154; P:rRNA modification; TAS:Reactome.
GO:0000179; F:rRNA (adenine-N6,N6-)-dimethyltransferase activity; IMP:UniProtKB.
GO:0000184; P:nuclear-transcribed mRNA catabolic process, nonsense-mediated decay; TAS:UniProtKB.
GO:0000213; F:tRNA-intron endonuclease activity; IBA:GO_Central.
GO:0000214; C:tRNA-intron endonuclease complex; IEA:InterPro.
GO:0000215; F:tRNA 2'-phosphotransferase activity; TAS:UniProtKB.
GO:0000288; P:nuclear-transcribed mRNA catabolic process, deadenylation-dependent decay; TAS:UniProtKB.
GO:0000289; P:nuclear-transcribed mRNA poly(A) tail shortening; TAS:Reactome.
GO:0000290; P:deadenylation-dependent decapping of nuclear-transcribed mRNA; IEA:Ensembl.
GO:0000291; P:nuclear-transcribed mRNA catabolic process, exonucleolytic; IMP:UniProtKB.
GO:0000294; P:nuclear-transcribed mRNA catabolic process, endonucleolytic cleavage-dependent decay; ISS:UniProtKB.
GO:0000339; F:RNA cap binding; TAS:UniProtKB.
GO:0000340; F:RNA 7-methylguanosine cap binding; IMP:UniProtKB.
GO:0000375; P:RNA splicing, via transesterification reactions; TAS:UniProtKB.
GO:0000379; P:tRNA-type intron splice site recognition and cleavage; IBA:GO_Central.
GO:0000380; P:alternative mRNA splicing, via spliceosome; ISS:UniProtKB.
GO:0000381; P:regulation of alternative mRNA splicing, via spliceosome; ISS:UniProtKB.
GO:0000389; P:mRNA 3'-splice site recognition; TAS:HGNC-UCL.
GO:0000395; P:mRNA 5'-splice site recognition; IDA:UniProtKB.
GO:0000398; P:mRNA splicing, via spliceosome; TAS:UniProtKB.
GO:0000430; P:regulation of transcription from RNA polymerase II promoter by glucose; IC:BHF-UCL.
GO:0000432; P:positive regulation of transcription from RNA polymerase II promoter by glucose; ISS:BHF-UCL.
GO:0000435; P:positive regulation of transcription from RNA polymerase II promoter by galactose; IDA:UniProtKB.
GO:0000447; P:endonucleolytic cleavage in ITS1 to separate SSU-rRNA from 5.8S rRNA and LSU-rRNA from tricistronic rRNA transcript (SSU-rRNA, 5.8S rRNA, LSU-rRNA); ISS:UniProtKB.
GO:0000448; P:cleavage in ITS2 between 5.8S rRNA and LSU-rRNA of tricistronic rRNA transcript (SSU-rRNA, 5.8S rRNA, LSU-rRNA); IEA:Ensembl.
GO:0000451; P:rRNA 2'-O-methylation; TAS:Reactome.
GO:0000453; P:enzyme-directed rRNA 2'-O-methylation; IEA:UniProtKB-UniRule.
GO:0000454; P:snoRNA guided rRNA pseudouridine synthesis; ISS:BHF-UCL.
GO:0000455; P:enzyme-directed rRNA pseudouridine synthesis; IMP:UniProtKB.
GO:0000460; P:maturation of 5.8S rRNA; IMP:UniProtKB.
GO:0000461; P:endonucleolytic cleavage to generate mature 3'-end of SSU-rRNA from (SSU-rRNA, 5.8S rRNA, LSU-rRNA); IBA:GO_Central.
GO:0000462; P:maturation of SSU-rRNA from tricistronic rRNA transcript (SSU-rRNA, 5.8S rRNA, LSU-rRNA); ISS:UniProtKB.
GO:0000463; P:maturation of LSU-rRNA from tricistronic rRNA transcript (SSU-rRNA, 5.8S rRNA, LSU-rRNA); IMP:UniProtKB.
GO:0000466; P:maturation of 5.8S rRNA from tricistronic rRNA transcript (SSU-rRNA, 5.8S rRNA, LSU-rRNA); IMP:UniProtKB.
GO:0000467; P:exonucleolytic trimming to generate mature 3'-end of 5.8S rRNA from tricistronic rRNA transcript (SSU-rRNA, 5.8S rRNA, LSU-rRNA); IBA:GO_Central.
GO:0000469; P:cleavage involved in rRNA processing; IEA:InterPro.
GO:0000470; P:maturation of LSU-rRNA; IBA:GO_Central.
GO:0000472; P:endonucleolytic cleavage to generate mature 5'-end of SSU-rRNA from (SSU-rRNA, 5.8S rRNA, LSU-rRNA); ISS:UniProtKB.
GO:0000480; P:endonucleolytic cleavage in 5'-ETS of tricistronic rRNA transcript (SSU-rRNA, 5.8S rRNA, LSU-rRNA); ISS:UniProtKB.
GO:0000481; P:maturation of 5S rRNA; IBA:GO_Central.
GO:0000494; P:box C/D snoRNA 3'-end processing; IBA:GO_Central.
GO:0000495; P:box H/ACA snoRNA 3'-end processing; IDA:UniProtKB.
GO:0000900; F:translation repressor activity, mRNA regulatory element binding; NAS:UniProtKB.
GO:0000956; P:nuclear-transcribed mRNA catabolic process; IMP:UniProtKB.
GO:0000957; P:mitochondrial RNA catabolic process; IDA:UniProtKB.
GO:0000958; P:mitochondrial mRNA catabolic process; IMP:UniProtKB.
GO:0000959; P:mitochondrial RNA metabolic process; IMP:UniProtKB.
GO:0000961; P:negative regulation of mitochondrial RNA catabolic process; IEA:Ensembl.
GO:0000962; P:positive regulation of mitochondrial RNA catabolic process; IDA:UniProtKB.
GO:0000963; P:mitochondrial RNA processing; IMP:UniProtKB.
GO:0000964; P:mitochondrial RNA 5'-end processing; IMP:UniProtKB.
GO:0000965; P:mitochondrial RNA 3'-end processing; IMP:UniProtKB.
GO:0000966; P:RNA 5'-end processing; ISS:UniProtKB.
GO:0000971; P:tRNA exon ligation utilizing 2',3' cyclic phosphate of 5'-exon as source of linkage phosphate; IBA:GO_Central.
GO:0000972; P:transcription-dependent tethering of RNA polymerase II gene DNA at nuclear periphery; ISS:BHF-UCL.

GO:0000973; P:posttranscriptional tethering of RNA polymerase II gene DNA at nuclear periphery; IBA:GO_Central.
GO:0000977; F:RNA polymerase II regulatory region sequence-specific DNA binding; ISS:UniProtKB.
GO:0000978; F:RNA polymerase II cis-regulatory region sequence-specific DNA binding; ISS:UniProtKB.
GO:0000979; F:RNA polymerase II core promoter sequence-specific DNA binding; ISS:UniProtKB.
GO:0000981; F:DNA-binding transcription factor activity, RNA polymerase II-specific; TAS:ProtInc.
GO:0000993; F:RNA polymerase II complex binding; ISS:UniProtKB.
GO:0000994; F:RNA polymerase III core binding; IBA:GO_Central.
GO:0000995; F:RNA polymerase III general transcription initiation factor activity; IMP:UniProtKB.
GO:0001002; F:RNA polymerase III type 1 promoter sequence-specific DNA binding; IBA:GO_Central.
GO:0001003; F:RNA polymerase III type 2 promoter sequence-specific DNA binding; IBA:GO_Central.
GO:0001004; F:RNA polymerase III transcription regulator recruiting activity; IEA:InterPro.
GO:0001006; F:RNA polymerase III type 3 promoter sequence-specific DNA binding; IBA:GO_Central.
GO:0001010; F:RNA polymerase II sequence-specific DNA-binding transcription factor recruiting activity; ISS:BHF-UCL.
GO:0001012; F:RNA polymerase II regulatory region DNA binding; IEA:Ensembl.
GO:0001013; F:RNA polymerase I regulatory region DNA binding; IDA:UniProtKB.
GO:0001016; F:RNA polymerase III regulatory region DNA binding; IDA:UniProtKB.
GO:0001030; F:RNA polymerase III type 1 promoter DNA binding; IDA:UniProtKB.
GO:0001031; F:RNA polymerase III type 2 promoter DNA binding; IDA:UniProtKB.
GO:0001032; F:RNA polymerase III type 3 promoter DNA binding; IDA:UniProtKB.
GO:0001042; F:RNA polymerase I core binding; ISS:UniProtKB.
GO:0001054; F:RNA polymerase I activity; IMP:ParkinsonsUK-UCL.
GO:0001055; F:RNA polymerase II activity; IEA:InterPro.
GO:0001069; F:regulatory region RNA binding; IEA:Ensembl.
GO:0001080; P:nitrogen catabolite activation of transcription from RNA polymerase II promoter; IC:BHF-UCL.
GO:0001085; F:RNA polymerase II transcription factor binding; ISS:BHF-UCL.
GO:0001091; F:RNA polymerase II general transcription initiation factor binding; IPI:ParkinsonsUK-UCL.
GO:0001099; F:basal RNA polymerase II transcription machinery binding; ISS:UniProtKB.
GO:0001102; F:RNA polymerase II activating transcription factor binding; TAS:BHF-UCL.
GO:0001103; F:RNA polymerase II repressing transcription factor binding; ISS:BHF-UCL.
GO:0001113; P:transcriptional open complex formation at RNA polymerase II promoter; IBA:GO_Central.
GO:0001135; F:RNA polymerase II transcription regulator recruiting activity; IBA:GO_Central.
GO:0001139; F:RNA polymerase II complex recruiting activity; IBA:GO_Central.
GO:0001162; F:RNA polymerase II intronic transcription regulatory region sequence-specific DNA binding; IEA:Ensembl.
GO:0001164; F:RNA polymerase I core promoter sequence-specific DNA binding; IMP:UniProtKB.
GO:0001165; F:RNA polymerase I cis-regulatory region sequence-specific DNA binding; IDA:UniProtKB.
GO:0001172; P:transcription, RNA-templated; IDA:BHF-UCL.
GO:0001174; P:transcriptional start site selection at RNA polymerase II promoter; IMP:UniProtKB.
GO:0001179; F:RNA polymerase I general transcription initiation factor binding; IEA:Ensembl.
GO:0001181; F:RNA polymerase I general transcription initiation factor activity; IBA:GO_Central.
GO:0001188; P:RNA polymerase I preinitiation complex assembly; IDA:UniProtKB.
GO:0001193; P:maintenance of transcriptional fidelity during DNA-templated transcription elongation from RNA polymerase II promoter; IBA:GO_Central.
GO:0001225; F:RNA polymerase II transcription coactivator binding; IPI:ARUK-UCL.
GO:0001226; F:RNA polymerase II transcription corepressor binding; IDA:UniProtKB.
GO:0001227; F:DNA-binding transcription repressor activity, RNA polymerase II-specific; NAS:BHF-UCL.
GO:0001228; F:DNA-binding transcription activator activity, RNA polymerase II-specific; ISS:UniProtKB.
GO:0001510; P:RNA methylation; ISS:UniProtKB.
GO:0001680; P:tRNA 3'-terminal CCA addition; IDA:UniProtKB.
GO:0001682; P:tRNA 5'-leader removal; IGI:CAFA.
GO:0001734; F:mRNA (N6-adenosine)-methyltransferase activity; IDA:UniProtKB.
GO:0002098; P:tRNA wobble uridine modification; NAS:UniProtKB.
GO:0002100; P:tRNA wobble adenosine to inosine editing; IBA:GO_Central.
GO:0002101; P:tRNA wobble cytosine modification; IDA:UniProtKB.
GO:0002127; P:tRNA wobble base cytosine methylation; IDA:UniProtKB.
GO:0002128; P:tRNA nucleoside ribose methylation; IEA:UniProtKB-UniRule.
GO:0002143; P:tRNA wobble position uridine thiolation; IBA:GO_Central.
GO:0002144; C:cytosolic tRNA wobble base thiouridylase complex; IBA:GO_Central.
GO:0002151; F:G-quadruplex RNA binding; ISS:UniProtKB.
GO:0002153; F:steroid receptor RNA activator RNA binding; IDA:UniProtKB.
GO:0002161; F:aminoacyl-tRNA editing activity; IEA:InterPro.
GO:0002192; P:IRES-dependent translational initiation of linear mRNA; IEA:Ensembl.
GO:0002196; F:Ser-tRNA(Ala) hydrolase activity; ISS:UniProtKB.
GO:0002926; P:tRNA wobble base 5-methoxycarbonylmethyl-2-thiouridinylation; IBA:GO_Central.
GO:0002939; P:tRNA N1-guanine methylation; IBA:GO_Central.
GO:0002940; P:tRNA N2-guanine methylation; IBA:GO_Central.
GO:0002943; P:tRNA dihydrouridine synthesis; IDA:UniProtKB.
GO:0002946; P:tRNA C5-cytosine methylation; IDA:UniProtKB.
GO:0002949; P:tRNA threonylcarbamoyladenosine modification; IEA:UniProtKB-UniRule.
GO:0003256; P:regulation of transcription from RNA polymerase II promoter involved in myocardial precursor cell

differentiation; ISS:BHF-UCL.
GO:0003257; P:positive regulation of transcription from RNA polymerase II promoter involved in myocardial precursor cell differentiation; ISS:BHF-UCL.
GO:0003721; F:telomerase RNA reverse transcriptase activity; IDA:BHF-UCL.
GO:0003723; F:RNA binding; TAS:UniProtKB.
GO:0003724; F:RNA helicase activity; TAS:ProtInc.
GO:0003725; F:double-stranded RNA binding; TAS:ProtInc.
GO:0003726; F:double-stranded RNA adenosine deaminase activity; IDA:MGI.
GO:0003727; F:single-stranded RNA binding; TAS:ProtInc.
GO:0003729; F:mRNA binding; TAS:UniProtKB.
GO:0003730; F:mRNA 3'-UTR binding; TAS:ProtInc.
GO:0003899; F:DNA-directed 5'-3' RNA polymerase activity; TAS:ProtInc.
GO:0003963; F:RNA-3'-phosphate cyclase activity; IBA:GO_Central.
GO:0003964; F:RNA-directed DNA polymerase activity; IEA:UniProtKB-KW.
GO:0003968; F:RNA-directed 5'-3' RNA polymerase activity; IEA:UniProtKB-KW.
GO:0003972; F:RNA ligase (ATP) activity; IDA:UniProtKB.
GO:0004045; F:aminoacyl-tRNA hydrolase activity; IMP:CAFA.
GO:0004479; F:methionyl-tRNA formyltransferase activity; IBA:GO_Central.
GO:0004482; F:mRNA (guanine-N7-)-methyltransferase activity; IDA:UniProtKB.
GO:0004483; F:mRNA (nucleoside-2'-O-)-methyltransferase activity; IDA:UniProtKB.
GO:0004484; F:mRNA guanylyltransferase activity; IDA:UniProtKB.
GO:0004523; F:RNA-DNA hybrid ribonuclease activity; TAS:UniProtKB.
GO:0004549; F:tRNA-specific ribonuclease activity; EXP:Reactome.
GO:0004809; F:tRNA (guanine-N2-)-methyltransferase activity; IBA:GO_Central.
GO:0004813; F:alanine-tRNA ligase activity; IMP:BHF-UCL.
GO:0004814; F:arginine-tRNA ligase activity; IEA:InterPro.
GO:0004815; F:aspartate-tRNA ligase activity; IDA:UniProtKB.
GO:0004816; F:asparagine-tRNA ligase activity; ISS:UniProtKB.
GO:0004817; F:cysteine-tRNA ligase activity; IDA:UniProtKB.
GO:0004818; F:glutamate-tRNA ligase activity; TAS:Reactome.
GO:0004819; F:glutamine-tRNA ligase activity; IDA:UniProtKB.
GO:0004820; F:glycine-tRNA ligase activity; IDA:UniProtKB.
GO:0004821; F:histidine-tRNA ligase activity; IDA:WormBase.
GO:0004822; F:isoleucine-tRNA ligase activity; IDA:UniProtKB.
GO:0004823; F:leucine-tRNA ligase activity; IDA:UniProtKB.
GO:0004824; F:lysine-tRNA ligase activity; IDA:UniProtKB.
GO:0004825; F:methionine-tRNA ligase activity; IDA:UniProtKB.
GO:0004826; F:phenylalanine-tRNA ligase activity; IEA:InterPro.
GO:0004827; F:proline-tRNA ligase activity; IDA:UniProtKB.
GO:0004828; F:serine-tRNA ligase activity; ISS:UniProtKB.
GO:0004829; F:threonine-tRNA ligase activity; ISS:UniProtKB.
GO:0004830; F:tryptophan-tRNA ligase activity; IMP:UniProtKB.
GO:0004831; F:tyrosine-tRNA ligase activity; IDA:BHF-UCL.
GO:0004832; F:valine-tRNA ligase activity; IDA:UniProtKB.
GO:0005665; C:RNA polymerase II, core complex; IEA:InterPro.
GO:0005666; C:RNA polymerase III complex; IDA:UniProtKB.
GO:0005668; C:RNA polymerase transcription factor SL1 complex; IEA:InterPro.
GO:0005736; C:RNA polymerase I complex; IBA:GO_Central.
GO:0005845; C:mRNA cap binding complex; ISS:UniProtKB.
GO:0005847; C:mRNA cleavage and polyadenylation specificity factor complex; IEA:Ensembl.
GO:0005848; C:mRNA cleavage stimulating factor complex; IBA:GO_Central.
GO:0005849; C:mRNA cleavage factor complex; IEA:UniProtKB-UniRule.
GO:0006269; P:DNA replication, synthesis of RNA primer; TAS:ProtInc.
GO:0006278; P:RNA-dependent DNA biosynthetic process; TAS:Reactome.
GO:0006356; P:regulation of transcription by RNA polymerase I; TAS:ProtInc.
GO:0006357; P:regulation of transcription by RNA polymerase II; TAS:UniProtKB.
GO:0006359; P:regulation of transcription by RNA polymerase III; TAS:UniProtKB.
GO:0006360; P:transcription by RNA polymerase I; TAS:ProtInc.
GO:0006361; P:transcription initiation from RNA polymerase I promoter; TAS:Reactome.
GO:0006362; P:transcription elongation from RNA polymerase I promoter; TAS:Reactome.
GO:0006363; P:termination of RNA polymerase I transcription; TAS:Reactome.
GO:0006364; P:rRNA processing; TAS:UniProtKB.
GO:0006366; P:transcription by RNA polymerase II; TAS:UniProtKB.
GO:0006367; P:transcription initiation from RNA polymerase II promoter; TAS:UniProtKB.
GO:0006368; P:transcription elongation from RNA polymerase II promoter; TAS:Reactome.
GO:0006369; P:termination of RNA polymerase II transcription; TAS:Reactome.
GO:0006370; P:7-methylguanosine mRNA capping; TAS:Reactome.
GO:0006376; P:mRNA splice site selection; TAS:ProtInc.
GO:0006378; P:mRNA polyadenylation; TAS:UniProtKB.

GO:0006379; P:mRNA cleavage; TAS:ProtInc.
GO:0006383; P:transcription by RNA polymerase III; TAS:ProtInc.
GO:0006384; P:transcription initiation from RNA polymerase III promoter; TAS:ProtInc.
GO:0006386; P:termination of RNA polymerase III transcription; IBA:GO_Central.
GO:0006388; P:tRNA splicing, via endonucleolytic cleavage and ligation; TAS:Reactome.
GO:0006396; P:RNA processing; TAS:UniProtKB.
GO:0006397; P:mRNA processing; TAS:UniProtKB.
GO:0006398; P:mRNA 3'-end processing by stem-loop binding and cleavage; ISS:UniProtKB.
GO:0006399; P:tRNA metabolic process; IBA:GO_Central.
GO:0006400; P:tRNA modification; TAS:UniProtKB.
GO:0006401; P:RNA catabolic process; TAS:ProtInc.
GO:0006402; P:mRNA catabolic process; TAS:ParkinsonsUK-UCL.
GO:0006403; P:RNA localization; IMP:MGI.
GO:0006404; P:RNA import into nucleus; IDA:UniProtKB.
GO:0006405; P:RNA export from nucleus; TAS:Reactome.
GO:0006406; P:mRNA export from nucleus; TAS:UniProtKB.
GO:0006407; P:rRNA export from nucleus; IMP:UniProtKB.
GO:0006408; P:snRNA export from nucleus; ISS:UniProtKB.
GO:0006409; P:tRNA export from nucleus; TAS:Reactome.
GO:0006418; P:tRNA aminoacylation for protein translation; TAS:Reactome.
GO:0006419; P:alanyl-tRNA aminoacylation; IEA:InterPro.
GO:0006420; P:arginyl-tRNA aminoacylation; IEA:InterPro.
GO:0006421; P:asparaginyl-tRNA aminoacylation; ISS:UniProtKB.
GO:0006422; P:aspartyl-tRNA aminoacylation; IBA:GO_Central.
GO:0006423; P:cysteinyl-tRNA aminoacylation; IDA:UniProtKB.
GO:0006424; P:glutamyl-tRNA aminoacylation; IEA:InterPro.
GO:0006425; P:glutaminyl-tRNA aminoacylation; IDA:UniProtKB.
GO:0006426; P:glycyl-tRNA aminoacylation; IBA:GO_Central.
GO:0006427; P:histidyl-tRNA aminoacylation; IDA:WormBase.
GO:0006428; P:isoleucyl-tRNA aminoacylation; IDA:UniProtKB.
GO:0006429; P:leucyl-tRNA aminoacylation; IDA:HGNC.
GO:0006430; P:lysyl-tRNA aminoacylation; IDA:UniProtKB.
GO:0006431; P:methionyl-tRNA aminoacylation; IDA:UniProtKB.
GO:0006432; P:phenylalanyl-tRNA aminoacylation; IDA:UniProtKB.
GO:0006433; P:prolyl-tRNA aminoacylation; IDA:UniProtKB.
GO:0006434; P:seryl-tRNA aminoacylation; ISS:UniProtKB.
GO:0006435; P:threonyl-tRNA aminoacylation; ISS:UniProtKB.
GO:0006436; P:tryptophanyl-tRNA aminoacylation; IBA:GO_Central.
GO:0006437; P:tyrosyl-tRNA aminoacylation; TAS:ProtInc.
GO:0006438; P:valyl-tRNA aminoacylation; IBA:GO_Central.
GO:0006990; P:positive regulation of transcription from RNA polymerase II promoter involved in unfolded protein response; ISS:UniProtKB.
GO:0008033; P:tRNA processing; TAS:ProtInc.
GO:0008097; F:5S rRNA binding; IMP:CAFA.
GO:0008135; F:translation factor activity, RNA binding; TAS:UniProtKB.
GO:0008173; F:RNA methyltransferase activity; TAS:Reactome.
GO:0008175; F:tRNA methyltransferase activity; IDA:UniProtKB.
GO:0008176; F:tRNA (guanine-N7-)-methyltransferase activity; IDA:UniProtKB.
GO:0008186; F:RNA-dependent ATPase activity; TAS:ProtInc.
GO:0008192; F:RNA guanylyltransferase activity; IDA:UniProtKB.
GO:0008193; F:tRNA guanylyltransferase activity; IDA:UniProtKB.
GO:0008251; F:tRNA-specific adenosine deaminase activity; IMP:UniProtKB.
GO:0008266; F:poly(U) RNA binding; TAS:ProtInc.
GO:0008298; P:intracellular mRNA localization; NAS:UniProtKB.
GO:0008312; F:7S RNA binding; TAS:ProtInc.
GO:0008334; P:histone mRNA metabolic process; TAS:Reactome.
GO:0008353; F:RNA polymerase II CTD heptapeptide repeat kinase activity; ISS:UniProtKB.
GO:0008380; P:RNA splicing; TAS:UniProtKB.
GO:0008419; F:RNA lariat debranching enzyme activity; IMP:UniProtKB.
GO:0008420; F:RNA polymerase II CTD heptapeptide repeat phosphatase activity; IMP:UniProtKB.
GO:0008479; F:queuine tRNA-ribosyltransferase activity; IEA:UniProtKB-UniRule.
GO:0008649; F:rRNA methyltransferase activity; IBA:GO_Central.
GO:0008650; F:rRNA (uridine-2'-O-)-methyltransferase activity; IBA:GO_Central.
GO:0008988; F:rRNA (adenine-N6-)-methyltransferase activity; IDA:UniProtKB.
GO:0009019; F:tRNA (guanine-N1-)-methyltransferase activity; IDA:CAFA.
GO:0009020; F:tRNA (guanosine-2'-O-)-methyltransferase activity; EXP:Reactome.
GO:0009299; P:mRNA transcription; IMP:UniProtKB.
GO:0009301; P:snRNA transcription; TAS:ProtInc.
GO:0009303; P:rRNA transcription; TAS:ProtInc.

GO:0009304; P:tRNA transcription; TAS:ProtInc.
GO:0009328; C:phenylalanine-tRNA ligase complex; IDA:UniProtKB.
GO:0009383; F:rRNA (cytosine-C5-)-methyltransferase activity; IBA:GO_Central.
GO:0009451; P:RNA modification; TAS:ProtInc.
GO:0009452; P:7-methylguanosine RNA capping; IEA:InterPro.
GO:0010501; P:RNA secondary structure unwinding; IDA:UniProtKB.
GO:0010526; P:negative regulation of transposition, RNA-mediated; IDA:UniProtKB.
GO:0010586; P:miRNA metabolic process; TAS:Reactome.
GO:0010587; P:miRNA catabolic process; IMP:UniProtKB.
GO:0010603; P:regulation of cytoplasmic mRNA processing body assembly; IDA:UniProtKB.
GO:0010606; P:positive regulation of cytoplasmic mRNA processing body assembly; IMP:UniProtKB.
GO:0010607; P:negative regulation of cytoplasmic mRNA processing body assembly; IEA:Ensembl.
GO:0010609; P:mRNA localization resulting in posttranscriptional regulation of gene expression; NAS:BHF-UCL.
GO:0010610; P:regulation of mRNA stability involved in response to stress; IMP:UniProtKB.
GO:0010767; P:regulation of transcription from RNA polymerase II promoter in response to UV-induced DNA damage; ISS:UniProtKB.
GO:0010768; P:negative regulation of transcription from RNA polymerase II promoter in response to UV-induced DNA damage; IMP:ParkinsonsUK-UCL.
GO:0010793; P:regulation of mRNA export from nucleus; ISS:UniProtKB.
GO:0016031; P:tRNA import into mitochondrion; IBA:GO_Central.
GO:0016070; P:RNA metabolic process; TAS:UniProtKB.
GO:0016071; P:mRNA metabolic process; NAS:UniProtKB.
GO:0016072; P:rRNA metabolic process; TAS:ARUK-UCL.
GO:0016073; P:snRNA metabolic process; IDA:UniProtKB.
GO:0016075; P:rRNA catabolic process; NAS:UniProtKB.
GO:0016076; P:snRNA catabolic process; ISS:UniProtKB.
GO:0016077; P:snoRNA catabolic process; IDA:UniProtKB.
GO:0016078; P:tRNA catabolic process; IDA:UniProtKB.
GO:0016180; P:snRNA processing; IMP:UniProtKB.
GO:0016246; P:RNA interference; NAS:UniProtKB.
GO:0016251; F:RNA polymerase II general transcription initiation factor activity; IMP:UniProtKB.
GO:0016300; F:tRNA (uracil) methyltransferase activity; IDA:UniProtKB.
GO:0016422; F:mRNA (2'-O-methyladenosine-N6-)-methyltransferase activity; IEA:InterPro.
GO:0016423; F:tRNA (guanine) methyltransferase activity; IBA:GO_Central.
GO:0016427; F:tRNA (cytosine) methyltransferase activity; ISS:UniProtKB.
GO:0016428; F:tRNA (cytosine-5-)-methyltransferase activity; IDA:UniProtKB.
GO:0016429; F:tRNA (adenine-N1-)-methyltransferase activity; IEA:InterPro.
GO:0016430; F:tRNA (adenine-N6-)-methyltransferase activity; IDA:UniProtKB.
GO:0016433; F:rRNA (adenine) methyltransferase activity; IMP:UniProtKB.
GO:0016435; F:rRNA (guanine) methyltransferase activity; IMP:UniProtKB.
GO:0016479; P:negative regulation of transcription by RNA polymerase I; IDA:CACAO.
GO:0016480; P:negative regulation of transcription by RNA polymerase III; IDA:UniProtKB.
GO:0016556; P:mRNA modification; IEA:InterPro.
GO:0016591; C:RNA polymerase II, holoenzyme; IEA:InterPro.
GO:0016973; P:poly(A)+ mRNA export from nucleus; IMP:UniProtKB.
GO:0017055; P:negative regulation of RNA polymerase II transcriptional preinitiation complex assembly; IDA:UniProtKB.
GO:0017069; F:snRNA binding; IDA:UniProtKB.
GO:0017070; F:U6 snRNA binding; NAS:UniProtKB.
GO:0017101; C:aminoacyl-tRNA synthetase multienzyme complex; IDA:UniProtKB.
GO:0017130; F:poly(C) RNA binding; IDA:UniProtKB.
GO:0017150; F:tRNA dihydrouridine synthase activity; IDA:UniProtKB.
GO:0017151; F:DEAD/H-box RNA helicase binding; TAS:UniProtKB.
GO:0019074; P:viral RNA genome packaging; IMP:CACAO.
GO:0019185; C:snRNA-activating protein complex; IDA:UniProtKB.
GO:0019843; F:rRNA binding; TAS:UniProtKB.
GO:0021882; P:regulation of transcription from RNA polymerase II promoter involved in forebrain neuron fate commitment; IEA:Ensembl.
GO:0021912; P:regulation of transcription from RNA polymerase II promoter involved in spinal cord motor neuron fate specification; IEA:Ensembl.
GO:0021913; P:regulation of transcription from RNA polymerase II promoter involved in ventral spinal cord interneuron specification; IEA:Ensembl.
GO:0021918; P:regulation of transcription from RNA polymerase II promoter involved in somatic motor neuron fate commitment; IEA:Ensembl.
GO:0021920; P:regulation of transcription from RNA polymerase II promoter involved in spinal cord association neuron specification; IEA:Ensembl.
GO:0030422; P:production of siRNA involved in RNA interference; TAS:Reactome.
GO:0030423; P:targeting of mRNA for destruction involved in RNA interference; IMP:UniProtKB.
GO:0030488; P:tRNA methylation; ISS:UniProtKB.
GO:0030490; P:maturation of SSU-rRNA; ISS:UniProtKB.

GO:0030515; F:snoRNA binding; ISS:UniProtKB.
GO:0030619; F:U1 snRNA binding; IDA:UniProtKB.
GO:0030620; F:U2 snRNA binding; IEA:Ensembl.
GO:0030621; F:U4 snRNA binding; IDA:UniProtKB.
GO:0030622; F:U4atac snRNA binding; IDA:UniProtKB.
GO:0030623; F:U5 snRNA binding; IBA:GO_Central.
GO:0030624; F:U6atac snRNA binding; IDA:UniProtKB.
GO:0030626; F:U12 snRNA binding; IBA:GO_Central.
GO:0030627; F:pre-mRNA 5'-splice site binding; IBA:GO_Central.
GO:0030628; F:pre-mRNA 3'-splice site binding; IDA:UniProtKB.
GO:0030629; F:U6 snRNA 3'-end binding; IDA:UniProtKB.
GO:0030697; F:S-adenosylmethionine-dependent tRNA (m5U54) methyltransferase activity; IEA:UniProtKB-EC.
GO:0030895; C:apolipoprotein B mRNA editing enzyme complex; TAS:HGNC-UCL.
GO:0030956; C:glutamyl-tRNA(Gln) amidotransferase complex; IDA:UniProtKB.
GO:0031047; P:gene silencing by RNA; ISS:UniProtKB.
GO:0031048; P:chromatin silencing by small RNA; IBA:GO_Central.
GO:0031053; P:primary miRNA processing; TAS:BHF-UCL.
GO:0031054; P:pre-miRNA processing; IMP:UniProtKB.
GO:0031086; P:nuclear-transcribed mRNA catabolic process, deadenylation-independent decay; ISS:UniProtKB.
GO:0031087; P:deadenylation-independent decapping of nuclear-transcribed mRNA; TAS:UniProtKB.
GO:0031118; P:rRNA pseudouridine synthesis; TAS:Reactome.
GO:0031119; P:tRNA pseudouridine synthesis; IMP:UniProtKB.
GO:0031120; P:snRNA pseudouridine synthesis; IBA:GO_Central.
GO:0031123; P:RNA 3'-end processing; IMP:UniProtKB.
GO:0031124; P:mRNA 3'-end processing; TAS:UniProtKB.
GO:0031125; P:rRNA 3'-end processing; ISS:UniProtKB.
GO:0031167; P:rRNA methylation; TAS:Reactome.
GO:0031379; C:RNA-directed RNA polymerase complex; IPI:BHF-UCL.
GO:0031380; C:nuclear RNA-directed RNA polymerase complex; IC:UniProtKB.
GO:0031439; P:positive regulation of mRNA cleavage; IDA:UniProtKB.
GO:0031440; P:regulation of mRNA 3'-end processing; IDA:UniProtKB.
GO:0031441; P:negative regulation of mRNA 3'-end processing; NAS:UniProtKB.
GO:0031442; P:positive regulation of mRNA 3'-end processing; IMP:UniProtKB.
GO:0031515; C:tRNA (m1A) methyltransferase complex; IBA:GO_Central.
GO:0031533; C:mRNA cap methyltransferase complex; IDA:UniProtKB.
GO:0031990; P:mRNA export from nucleus in response to heat stress; IDA:UniProtKB.
GO:0032197; P:transposition, RNA-mediated; IMP:UniProtKB.
GO:0032199; P:reverse transcription involved in RNA-mediated transposition; IDA:UniProtKB.
GO:0032574; F:5'-3' RNA helicase activity; IDA:UniProtKB.
GO:0032968; P:positive regulation of transcription elongation from RNA polymerase II promoter; ISS:UniProtKB.
GO:0033119; P:negative regulation of RNA splicing; IDA:UniProtKB.
GO:0033120; P:positive regulation of RNA splicing; IMP:UniProtKB.
GO:0033168; P:conversion of ds siRNA to ss siRNA involved in RNA interference; IMP:BHF-UCL.
GO:0033204; F:ribonuclease P RNA binding; IDA:UniProtKB.
GO:0033227; P:dsRNA transport; IMP:UniProtKB.
GO:0033592; F:RNA strand annealing activity; IEA:InterPro.
GO:0033677; F:DNA/RNA helicase activity; IDA:UniProtKB.
GO:0033678; F:5'-3' DNA/RNA helicase activity; IDA:BHF-UCL.
GO:0033679; F:3'-5' DNA/RNA helicase activity; IDA:UniProtKB.
GO:0033962; P:cytoplasmic mRNA processing body assembly; ISS:BHF-UCL.
GO:0033979; P:box H/ACA snoRNA metabolic process; IEA:Ensembl.
GO:0034062; F:5'-3' RNA polymerase activity; TAS:Reactome.
GO:0034227; P:tRNA thio-modification; NAS:UniProtKB.
GO:0034243; P:regulation of transcription elongation from RNA polymerase II promoter; ISS:UniProtKB.
GO:0034244; P:negative regulation of transcription elongation from RNA polymerase II promoter; ISS:UniProtKB.
GO:0034245; C:mitochondrial DNA-directed RNA polymerase complex; IBA:GO_Central.
GO:0034247; P:snoRNA splicing; IBA:GO_Central.
GO:0034337; P:RNA folding; IDA:UniProtKB.
GO:0034353; F:RNA pyrophosphohydrolase activity; ISS:UniProtKB.
GO:0034395; P:regulation of transcription from RNA polymerase II promoter in response to iron; IEA:Ensembl.
GO:0034402; P:recruitment of 3'-end processing factors to RNA polymerase II holoenzyme complex; IBA:GO_Central.
GO:0034427; P:nuclear-transcribed mRNA catabolic process, exonucleolytic, 3'-5'; IEA:InterPro.
GO:0034458; F:3'-5' RNA helicase activity; IDA:UniProtKB.
GO:0034472; P:snRNA 3'-end processing; IMP:UniProtKB.
GO:0034473; P:U1 snRNA 3'-end processing; IBA:GO_Central.
GO:0034474; P:U2 snRNA 3'-end processing; IBA:GO_Central.
GO:0034475; P:U4 snRNA 3'-end processing; IBA:GO_Central.
GO:0034476; P:U5 snRNA 3'-end processing; IBA:GO_Central.
GO:0034477; P:U6 snRNA 3'-end processing; IMP:UniProtKB.

GO:0034511; F:U3 snoRNA binding; IEA:InterPro.
GO:0034512; F:box C/D snoRNA binding; IEA:Ensembl.
GO:0034513; F:box H/ACA snoRNA binding; IPI:BHF-UCL.
GO:0034518; C:RNA cap binding complex; TAS:UniProtKB.
GO:0034584; F:piRNA binding; ISS:UniProtKB.
GO:0034587; P:piRNA metabolic process; ISS:UniProtKB.
GO:0035087; P:siRNA loading onto RISC involved in RNA interference; IDA:UniProtKB.
GO:0035194; P:posttranscriptional gene silencing by RNA; TAS:Reactome.
GO:0035195; P:gene silencing by miRNA; TAS:UniProtKB.
GO:0035196; P:production of miRNAs involved in gene silencing by miRNA; IMP:UniProtKB.
GO:0035197; F:siRNA binding; ISS:UniProtKB.
GO:0035198; F:miRNA binding; ISS:UniProtKB.
GO:0035278; P:miRNA mediated inhibition of translation; TAS:UniProtKB.
GO:0035279; P:mRNA cleavage involved in gene silencing by miRNA; IDA:UniProtKB.
GO:0035280; P:miRNA loading onto RISC involved in gene silencing by miRNA; IDA:MGI.
GO:0035281; P:pre-miRNA export from nucleus; IDA:BHF-UCL.
GO:0035513; P:oxidative RNA demethylation; IDA:UniProtKB.
GO:0035515; F:oxidative RNA demethylase activity; IMP:UniProtKB.
GO:0035553; P:oxidative single-stranded RNA demethylation; IDA:UniProtKB.
GO:0035600; P:tRNA methylthiolation; IBA:GO_Central.
GO:0035613; F:RNA stem-loop binding; ISS:UniProtKB.
GO:0035925; F:mRNA 3'-UTR AU-rich region binding; ISS:UniProtKB.
GO:0035927; P:RNA import into mitochondrion; IDA:UniProtKB.
GO:0035928; P:rRNA import into mitochondrion; IMP:UniProtKB.
GO:0035945; P:mitochondrial ncRNA surveillance; IMP:UniProtKB.
GO:0035946; P:mitochondrial mRNA surveillance; IMP:UniProtKB.
GO:0035947; P:regulation of gluconeogenesis by regulation of transcription from RNA polymerase II promoter; ISS:UniProtKB.
GO:0035948; P:positive regulation of gluconeogenesis by positive regulation of transcription from RNA polymerase II promoter; IEA:Ensembl.
GO:0036002; F:pre-mRNA binding; TAS:BHF-UCL.
GO:0036003; P:positive regulation of transcription from RNA polymerase II promoter in response to stress; ISS:UniProtKB.
GO:0036031; P:recruitment of mRNA capping enzyme to RNA polymerase II holoenzyme complex; IDA:UniProtKB.
GO:0036091; P:positive regulation of transcription from RNA polymerase II promoter in response to oxidative stress; IMP:UniProtKB.
GO:0036265; P:RNA (guanine-N7)-methylation; IBA:GO_Central.
GO:0036317; F:tyrosyl-RNA phosphodiesterase activity; IDA:UniProtKB.
GO:0036396; C:RNA N6-methyladenosine methyltransferase complex; IDA:UniProtKB.
GO:0036404; P:conversion of ds siRNA to ss siRNA; IMP:AgBase.
GO:0036416; P:tRNA stabilization; ISS:UniProtKB.
GO:0039689; P:negative stranded viral RNA replication; IEA:Ensembl.
GO:0039692; P:single stranded viral RNA replication via double stranded DNA intermediate; IDA:MGI.
GO:0039694; P:viral RNA genome replication; IMP:ParkinsonsUK-UCL.
GO:0040031; P:snRNA modification; IDA:UniProtKB.
GO:0042134; F:rRNA primary transcript binding; IDA:UniProtKB.
GO:0042245; P:RNA repair; IDA:UniProtKB.
GO:0042272; C:nuclear RNA export factor complex; NAS:UniProtKB.
GO:0042565; C:RNA nuclear export complex; IDA:BHF-UCL.
GO:0042779; P:tRNA 3'-trailer cleavage; IEA:InterPro.
GO:0042780; P:tRNA 3'-end processing; TAS:Reactome.
GO:0042781; F:3'-tRNA processing endoribonuclease activity; IBA:GO_Central.
GO:0042789; P:mRNA transcription by RNA polymerase II; TAS:BHF-UCL.
GO:0042790; P:nucleolar large rRNA transcription by RNA polymerase I; IBA:GO_Central.
GO:0042791; P:5S class rRNA transcription by RNA polymerase III; IC:HGNC-UCL.
GO:0042795; P:snRNA transcription by RNA polymerase II; TAS:Reactome.
GO:0042796; P:snRNA transcription by RNA polymerase III; IMP:UniProtKB.
GO:0042797; P:tRNA transcription by RNA polymerase III; IC:HGNC-UCL.
GO:0043039; P:tRNA aminoacylation; IDA:BHF-UCL.
GO:0043137; P:DNA replication, removal of RNA primer; IDA:UniProtKB.
GO:0043175; F:RNA polymerase core enzyme binding; IPI:UniProtKB.
GO:0043330; P:response to exogenous dsRNA; ISS:UniProtKB.
GO:0043331; P:response to dsRNA; IEA:Ensembl.
GO:0043484; P:regulation of RNA splicing; ISS:UniProtKB.
GO:0043488; P:regulation of mRNA stability; TAS:Reactome.
GO:0043489; P:RNA stabilization; ISS:BHF-UCL.
GO:0043527; C:tRNA methyltransferase complex; IDA:UniProtKB.
GO:0043618; P:regulation of transcription from RNA polymerase II promoter in response to stress; IDA:BHF-UCL.
GO:0043619; P:regulation of transcription from RNA polymerase II promoter in response to oxidative stress; ISS:BHF-UCL.
GO:0043629; P:ncRNA polyadenylation; IDA:UniProtKB.
GO:0043630; P:ncRNA polyadenylation involved in polyadenylation-dependent ncRNA catabolic process; IMP:BHF-UCL.

GO:0043631; P:RNA polyadenylation; ISS:UniProtKB.
GO:0043928; P:exonucleolytic catabolism of deadenylated mRNA; TAS:Reactome.
GO:0044377; F:RNA polymerase II cis-regulatory region sequence-specific DNA binding, bending; IEA:Ensembl.
GO:0044528; P:regulation of mitochondrial mRNA stability; IMP:UniProtKB.
GO:0044830; P:modulation by host of viral RNA genome replication; IMP:UniProtKB.
GO:0045091; P:regulation of single stranded viral RNA replication via double stranded DNA intermediate; IDA:UniProtKB.
GO:0045131; F:pre-mRNA branch point binding; IEA:InterPro.
GO:0045292; P:mRNA cis splicing, via spliceosome; IEA:InterPro.
GO:0045869; P:negative regulation of single stranded viral RNA replication via double stranded DNA intermediate; ISS:UniProtKB.
GO:0045870; P:positive regulation of single stranded viral RNA replication via double stranded DNA intermediate; IMP:UniProtKB.
GO:0045898; P:regulation of RNA polymerase II transcriptional preinitiation complex assembly; IDA:UniProtKB.
GO:0045899; P:positive regulation of RNA polymerase II transcriptional preinitiation complex assembly; IEA:Ensembl.
GO:0045943; P:positive regulation of transcription by RNA polymerase I; ISS:UniProtKB.
GO:0045944; P:positive regulation of transcription by RNA polymerase II; TAS:UniProtKB.
GO:0045945; P:positive regulation of transcription by RNA polymerase III; IMP:UniProtKB.
GO:0046778; P:modification by virus of host mRNA processing; TAS:Reactome.
GO:0046784; P:viral mRNA export from host cell nucleus; IDA:UniProtKB.
GO:0046831; P:regulation of RNA export from nucleus; ISS:UniProtKB.
GO:0046832; P:negative regulation of RNA export from nucleus; IDA:UniProtKB.
GO:0046833; P:positive regulation of RNA export from nucleus; ISS:UniProtKB.
GO:0048024; P:regulation of mRNA splicing, via spliceosome; TAS:UniProtKB.
GO:0048025; P:negative regulation of mRNA splicing, via spliceosome; ISS:UniProtKB.
GO:0048026; P:positive regulation of mRNA splicing, via spliceosome; ISS:UniProtKB.
GO:0048027; F:mRNA 5'-UTR binding; IMP:CAFA.
GO:0048254; P:snoRNA localization; IMP:UniProtKB.
GO:0048255; P:mRNA stabilization; TAS:UniProtKB.
GO:0050265; F:RNA uridylyltransferase activity; IDA:UniProtKB.
GO:0050560; F:aspartate-tRNA(Asn) ligase activity; IDA:BHF-UCL.
GO:0050561; F:glutamate-tRNA(Gln) ligase activity; IDA:UniProtKB.
GO:0050567; F:glutaminyl-tRNA synthase (glutamine-hydrolyzing) activity; IDA:UniProtKB.
GO:0050658; P:RNA transport; TAS:UniProtKB.
GO:0050684; P:regulation of mRNA processing; ISS:UniProtKB.
GO:0050779; P:RNA destabilization; ISS:UniProtKB.
GO:0051028; P:mRNA transport; ISS:UniProtKB.
GO:0051029; P:rRNA transport; IDA:UniProtKB.
GO:0051031; P:tRNA transport; IMP:UniProtKB.
GO:0051033; F:RNA transmembrane transporter activity; IEA:InterPro.
GO:0051123; P:RNA polymerase II preinitiation complex assembly; ISS:BHF-UCL.
GO:0051252; P:regulation of RNA metabolic process; NAS:UniProtKB.
GO:0051391; P:tRNA acetylation; IEA:UniProtKB-UniRule.
GO:0051500; F:D-tyrosyl-tRNA(Tyr) deacylase activity; IBA:GO_Central.
GO:0052381; F:tRNA dimethylallyltransferase activity; EXP:Reactome.
GO:0052666; F:tRNA (cytosine-2'-O-)-methyltransferase activity; EXP:Reactome.
GO:0052717; F:tRNA-specific adenosine-34 deaminase activity; IBA:GO_Central.
GO:0052718; C:tRNA-specific adenosine-34 deaminase complex; IBA:GO_Central.
GO:0052735; F:tRNA (cytosine-3-)-methyltransferase activity; IEA:Ensembl.
GO:0052905; F:tRNA (guanine(9)-N(1))-methyltransferase activity; IEA:UniProtKB-EC.
GO:0052906; F:tRNA (guanine(37)-N(1))-methyltransferase activity; IEA:UniProtKB-EC.
GO:0052907; F:23S rRNA (adenine(1618)-N(6))-methyltransferase activity; IBA:GO_Central.
GO:0052909; F:18S rRNA (adenine(1779)-N(6)/adenine(1780)-N(6))-dimethyltransferase activity; IEA:UniProtKB-EC.
GO:0052927; F:CTP:tRNA cytidylyltransferase activity; IEA:UniProtKB-EC.
GO:0052928; F:CTP:3'-cytidine-tRNA cytidylyltransferase activity; IEA:UniProtKB-EC.
GO:0052929; F:ATP:3'-cytidine-cytidine-tRNA adenylyltransferase activity; IDA:UniProtKB.
GO:0060212; P:negative regulation of nuclear-transcribed mRNA poly(A) tail shortening; IMP:UniProtKB.
GO:0060213; P:positive regulation of nuclear-transcribed mRNA poly(A) tail shortening; ISS:UniProtKB.
GO:0060260; P:regulation of transcription initiation from RNA polymerase II promoter; NAS:ParkinsonsUK-UCL.
GO:0060261; P:positive regulation of transcription initiation from RNA polymerase II promoter; ISS:BHF-UCL.
GO:0060633; P:negative regulation of transcription initiation from RNA polymerase II promoter; IMP:UniProtKB.
GO:0060735; P:regulation of eIF2 alpha phosphorylation by dsRNA; IDA:UniProtKB.
GO:0060807; P:regulation of transcription from RNA polymerase II promoter involved in definitive endodermal cell fate specification; ISS:BHF-UCL.
GO:0060964; P:regulation of gene silencing by miRNA; TAS:UniProtKB.
GO:0060965; P:negative regulation of gene silencing by miRNA; IMP:UniProtKB.
GO:0060994; P:regulation of transcription from RNA polymerase II promoter involved in kidney development; IEA:Ensembl.
GO:0061014; P:positive regulation of mRNA catabolic process; ISS:UniProtKB.
GO:0061015; P:snRNA import into nucleus; IMP:UniProtKB.
GO:0061157; P:mRNA destabilization; NAS:BHF-UCL.

GO:0061158; P:3'-UTR-mediated mRNA destabilization; ISS:UniProtKB.
GO:0061394; P:regulation of transcription from RNA polymerase II promoter in response to arsenic-containing substance; TAS:ParkinsonsUK-UCL.
GO:0061395; P:positive regulation of transcription from RNA polymerase II promoter in response to arsenic-containing substance; TAS:ParkinsonsUK-UCL.
GO:0061396; P:regulation of transcription from RNA polymerase II promoter in response to copper ion; IEA:Ensembl.
GO:0061400; P:positive regulation of transcription from RNA polymerase II promoter in response to calcium ion; IDA:UniProtKB.
GO:0061402; P:positive regulation of transcription from RNA polymerase II promoter in response to acidic pH; IEA:Ensembl.
GO:0061408; P:positive regulation of transcription from RNA polymerase II promoter in response to heat stress; IDA:UniProtKB.
GO:0061418; P:regulation of transcription from RNA polymerase II promoter in response to hypoxia; TAS:Reactome.
GO:0061419; P:positive regulation of transcription from RNA polymerase II promoter in response to hypoxia; ISS:UniProtKB.
GO:0061428; P:negative regulation of transcription from RNA polymerase II promoter in response to hypoxia; IMP:UniProtKB.
GO:0061614; P:pri-miRNA transcription by RNA polymerase II; ISS:UniProtKB.
GO:0061629; F:RNA polymerase II-specific DNA-binding transcription factor binding; ISS:BHF-UCL.
GO:0061632; F:RNA lariat debranching enzyme activator activity; IBA:GO_Central.
GO:0061712; F:tRNA (N(6)-L-threonylcarbamoyladenosine(37)-C(2))-methylthiotransferase; IEA:UniProtKB-EC.
GO:0061715; P:miRNA 2'-O-methylation; IDA:UniProtKB.
GO:0061752; F:telomeric repeat-containing RNA binding; IDA:BHF-UCL.
GO:0061953; F:mRNA (adenine-N1-)-methyltransferase activity; IDA:UniProtKB.
GO:0061987; P:negative regulation of transcription from RNA polymerase II promoter by glucose; ISS:BHF-UCL.
GO:0062103; P:double-stranded RNA biosynthetic process; IDA:ARUK-UCL.
GO:0062105; F:RNA 2'-O-methyltransferase activity; IDA:UniProtKB.
GO:0062152; F:mRNA (cytidine-5-)-methyltransferase activity; IDA:UniProtKB.
GO:0062153; F:C5-methylcytidine-containing RNA binding; IDA:UniProtKB.
GO:0070034; F:telomerase RNA binding; ISS:BHF-UCL.
GO:0070037; F:rRNA (pseudouridine) methyltransferase activity; IDA:UniProtKB.
GO:0070039; F:rRNA (guanosine-2'-O-)-methyltransferase activity; EXP:Reactome.
GO:0070042; F:rRNA (uridine-N3-)-methyltransferase activity; IBA:GO_Central.
GO:0070054; P:mRNA splicing, via endonucleolytic cleavage and ligation; IDA:UniProtKB.
GO:0070063; F:RNA polymerase binding; ISS:UniProtKB.
GO:0070127; P:tRNA aminoacylation for mitochondrial protein translation; IDA:UniProtKB.
GO:0070143; P:mitochondrial alanyl-tRNA aminoacylation; IMP:BHF-UCL.
GO:0070145; P:mitochondrial asparaginyl-tRNA aminoacylation; IDA:UniProtKB.
GO:0070150; P:mitochondrial glycyl-tRNA aminoacylation; IBA:GO_Central.
GO:0070158; P:mitochondrial seryl-tRNA aminoacylation; IBA:GO_Central.
GO:0070159; P:mitochondrial threonyl-tRNA aminoacylation; TAS:BHF-UCL.
GO:0070180; F:large ribosomal subunit rRNA binding; IBA:GO_Central.
GO:0070181; F:small ribosomal subunit rRNA binding; IEA:Ensembl.
GO:0070183; P:mitochondrial tryptophanyl-tRNA aminoacylation; IBA:GO_Central.
GO:0070184; P:mitochondrial tyrosyl-tRNA aminoacylation; IMP:BHF-UCL.
GO:0070329; P:tRNA seleno-modification; IBA:GO_Central.
GO:0070475; P:rRNA base methylation; IBA:GO_Central.
GO:0070476; P:rRNA (guanine-N7)-methylation; IMP:UniProtKB.
GO:0070478; P:nuclear-transcribed mRNA catabolic process, 3'-5' exonucleolytic nonsense-mediated decay; IBA:GO_Central.
GO:0070481; P:nuclear-transcribed mRNA catabolic process, non-stop decay; IEA:InterPro.
GO:0070525; P:tRNA threonylcarbamoyladenosine metabolic process; IBA:GO_Central.
GO:0070551; F:endoribonuclease activity, cleaving siRNA-paired mRNA; IDA:UniProtKB.
GO:0070651; P:nonfunctional rRNA decay; IBA:GO_Central.
GO:0070681; P:glutaminyl-tRNAGln biosynthesis via transamidation; IDA:UniProtKB.
GO:0070816; P:phosphorylation of RNA polymerase II C-terminal domain; IMP:UniProtKB.
GO:0070860; C:RNA polymerase I core factor complex; IDA:UniProtKB.
GO:0070878; F:primary miRNA binding; ISS:BHF-UCL.
GO:0070883; F:pre-miRNA binding; ISS:UniProtKB.
GO:0070898; P:RNA polymerase III preinitiation complex assembly; IEA:GOC.
GO:0070899; P:mitochondrial tRNA wobble uridine modification; IBA:GO_Central.
GO:0070900; P:mitochondrial tRNA modification; TAS:Reactome.
GO:0070901; P:mitochondrial tRNA methylation; TAS:Reactome.
GO:0070902; P:mitochondrial tRNA pseudouridine synthesis; TAS:Reactome.
GO:0070922; P:small RNA loading onto RISC; IMP:UniProtKB.
GO:0070934; P:CRD-mediated mRNA stabilization; IMP:UniProtKB.
GO:0070935; P:3'-UTR-mediated mRNA stabilization; TAS:UniProtKB.
GO:0070937; C:CRD-mediated mRNA stability complex; IDA:UniProtKB.
GO:0070940; P:dephosphorylation of RNA polymerase II C-terminal domain; IMP:UniProtKB.
GO:0070966; P:nuclear-transcribed mRNA catabolic process, no-go decay; IMP:UniProtKB.
GO:0071008; C:U2-type post-mRNA release spliceosomal complex; IDA:UniProtKB.
GO:0071014; C:post-mRNA release spliceosomal complex; IBA:GO_Central.
GO:0071025; P:RNA surveillance; IEA:InterPro.

GO:0071028; P:nuclear mRNA surveillance; ISS:UniProtKB.
GO:0071033; P:nuclear retention of pre-mRNA at the site of transcription; IBA:GO_Central.
GO:0071035; P:nuclear polyadenylation-dependent rRNA catabolic process; IMP:UniProtKB.
GO:0071036; P:nuclear polyadenylation-dependent snoRNA catabolic process; IBA:GO_Central.
GO:0071037; P:nuclear polyadenylation-dependent snRNA catabolic process; IBA:GO_Central.
GO:0071038; P:nuclear polyadenylation-dependent tRNA catabolic process; IBA:GO_Central.
GO:0071042; P:nuclear polyadenylation-dependent mRNA catabolic process; IDA:UniProtKB.
GO:0071044; P:histone mRNA catabolic process; IMP:UniProtKB.
GO:0071045; P:nuclear histone mRNA catabolic process; IMP:CAFA.
GO:0071048; P:nuclear retention of unspliced pre-mRNA at the site of transcription; IMP:UniProtKB.
GO:0071049; P:nuclear retention of pre-mRNA with aberrant 3'-ends at the site of transcription; IBA:GO_Central.
GO:0071050; P:snoRNA polyadenylation; IDA:UniProtKB.
GO:0071051; P:polyadenylation-dependent snoRNA 3'-end processing; IDA:UniProtKB.
GO:0071076; P:RNA 3' uridylation; ISS:UniProtKB.
GO:0071164; F:RNA trimethylguanosine synthase activity; IDA:BHF-UCL.
GO:0071204; C:histone pre-mRNA 3'end processing complex; ISS:UniProtKB.
GO:0071207; F:histone pre-mRNA stem-loop binding; ISS:UniProtKB.
GO:0071208; F:histone pre-mRNA DCP binding; ISS:UniProtKB.
GO:0071209; F:U7 snRNA binding; IPI:BHF-UCL.
GO:0071359; P:cellular response to dsRNA; IMP:UniProtKB.
GO:0071360; P:cellular response to exogenous dsRNA; TAS:BHF-UCL.
GO:0071424; F:rRNA (cytosine-N4-)-methyltransferase activity; IBA:GO_Central.
GO:0071528; P:tRNA re-export from nucleus; IBA:GO_Central.
GO:0071951; P:conversion of methionyl-tRNA to N-formyl-methionyl-tRNA; IBA:GO_Central.
GO:0072368; P:regulation of lipid transport by negative regulation of transcription from RNA polymerase II promoter; IDA:BHF-UCL.
GO:0072369; P:regulation of lipid transport by positive regulation of transcription from RNA polymerase II promoter; IMP:BHF-UCL.
GO:0072669; C:tRNA-splicing ligase complex; IDA:UniProtKB.
GO:0072684; P:mitochondrial tRNA 3'-trailer cleavage, endonucleolytic; IMP:UniProtKB.
GO:0080009; P:mRNA methylation; IMP:UniProtKB.
GO:0090065; P:regulation of production of siRNA involved in RNA interference; IEA:InterPro.
GO:0090366; P:positive regulation of mRNA modification; IEA:Ensembl.
GO:0090501; P:RNA phosphodiester bond hydrolysis; ISS:UniProtKB.
GO:0090502; P:RNA phosphodiester bond hydrolysis, endonucleolytic; IEA:GOC.
GO:0090503; P:RNA phosphodiester bond hydrolysis, exonucleolytic; ISS:BHF-UCL.
GO:0090571; C:RNA polymerase II transcription repressor complex; IEA:Ensembl.
GO:0090575; C:RNA polymerase II transcription factor complex; ISS:BHF-UCL.
GO:0090615; P:mitochondrial mRNA processing; IMP:UniProtKB.
GO:0090624; F:endoribonuclease activity, cleaving miRNA-paired mRNA; IMP:UniProtKB.
GO:0090625; P:mRNA cleavage involved in gene silencing by siRNA; IDA:BHF-UCL.
GO:0090646; P:mitochondrial tRNA processing; TAS:Reactome.
GO:0090666; P:scaRNA localization to Cajal body; IMP:BHF-UCL.
GO:0090669; P:telomerase RNA stabilization; IMP:BHF-UCL.
GO:0090671; P:telomerase RNA localization to Cajal body; IMP:BHF-UCL.
GO:0097056; P:selenocysteinyl-tRNA(Sec) biosynthetic process; IEA:UniProtKB-UniPathway.
GO:0097157; F:pre-mRNA intronic binding; ISS:UniProtKB.
GO:0097158; F:pre-mRNA intronic pyrimidine-rich binding; IDA:UniProtKB.
GO:0097201; P:negative regulation of transcription from RNA polymerase II promoter in response to stress; ISS:UniProtKB.
GO:0097222; P:mitochondrial mRNA polyadenylation; IMP:UniProtKB.
GO:0097309; P:cap1 mRNA methylation; IDA:UniProtKB.
GO:0097310; P:cap2 mRNA methylation; IDA:UniProtKB.
GO:0097322; F:7SK snRNA binding; IEA:Ensembl.
GO:0097694; P:establishment of RNA localization to telomere; IMP:BHF-UCL.
GO:0097745; P:mitochondrial tRNA 5'-end processing; IDA:UniProtKB.
GO:0098680; F:template-free RNA nucleotidyltransferase; IDA:BHF-UCL.
GO:0098781; P:ncRNA transcription; IEA:Ensembl.
GO:0098787; P:mRNA cleavage involved in mRNA processing; IDA:ParkinsonsUK-UCL.
GO:0098789; P:pre-mRNA cleavage required for polyadenylation; IMP:UniProtKB.
GO:0098808; F:mRNA cap binding; IPI:ParkinsonsUK-UCL.
GO:0099122; F:RNA polymerase II C-terminal domain binding; ISS:UniProtKB.
GO:0101030; P:tRNA-guanine transglycosylation; IDA:UniProtKB.
GO:0102264; F:tRNA-dihydrouridine20 synthase activity; IEA:UniProtKB-EC.
GO:0102521; F:tRNA-4-demethylwyosine synthase activity; IEA:UniProtKB-EC.
GO:0102522; F:tRNA 4-demethylwyosine alpha-amino-alpha-carboxypropyltransferase activity; IEA:UniProtKB-EC.
GO:0102524; F:tRNAPhe (7-(3-amino-3-carboxypropyl)wyosine37-C2)-hydroxylase activity; IEA:UniProtKB-EC.
GO:0106004; P:tRNA (guanine-N7)-methylation; IEA:GOC.
GO:0106005; P:RNA 5'-cap (guanine-N7)-methylation; IEA:InterPro.
GO:0106029; F:tRNA pseudouridine synthase activity; IEA:UniProtKB-EC.

GO:0106050; F:tRNA 2'-O-methyltransferase activity; IEA:InterPro.
GO:0106074; P:aminoacyl-tRNA metabolism involved in translational fidelity; IDA:UniProtKB.
GO:0106105; F:Ala-tRNA(Thr) hydrolase activity; IDA:UniProtKB.
GO:0106162; F:mRNA N-acetyltransferase activity; IDA:UniProtKB.
GO:0110008; P:ncRNA deadenylation; IMP:BHF-UCL.
GO:0110104; P:mRNA alternative polyadenylation; IMP:UniProtKB.
GO:0110152; F:RNA NAD-cap (NAD-forming) hydrolase activity; ISS:UniProtKB.
GO:0110153; F:RNA NAD-cap (NMN-forming) hydrolase activity; ISS:UniProtKB.
GO:0120048; F:U6 snRNA (adenine-(43)-N(6))-methyltransferase activity; IDA:UniProtKB.
GO:0120049; P:snRNA (adenine-N6)-methylation; IDA:UniProtKB.
GO:0140262; F:mRNA cap binding complex binding; IEA:Ensembl.
GO:1900153; P:positive regulation of nuclear-transcribed mRNA catabolic process, deadenylation-dependent decay; ISS:UniProtKB.
GO:1900260; P:negative regulation of RNA-directed 5'-3' RNA polymerase activity; IDA:AgBase.
GO:1900363; P:regulation of mRNA polyadenylation; IMP:UniProtKB.
GO:1900364; P:negative regulation of mRNA polyadenylation; IMP:UniProtKB.
GO:1900365; P:positive regulation of mRNA polyadenylation; ISS:UniProtKB.
GO:1900369; P:negative regulation of RNA interference; IEA:Ensembl.
GO:1900370; P:positive regulation of RNA interference; IDA:BHF-UCL.
GO:1900387; P:negative regulation of cell-cell adhesion by negative regulation of transcription from RNA polymerase II promoter; IMP:BHF-UCL.
GO:1900413; P:positive regulation of phospholipid biosynthetic process by positive regulation of transcription from RNA polymerase II promoter; TAS:ParkinsonsUK-UCL.
GO:1900477; P:negative regulation of G1/S transition of mitotic cell cycle by negative regulation of transcription from RNA polymerase II promoter; IDA:BHF-UCL.
GO:1901227; P:negative regulation of transcription from RNA polymerase II promoter involved in heart development; ISS:BHF-UCL.
GO:1901228; P:positive regulation of transcription from RNA polymerase II promoter involved in heart development; ISS:BHF-UCL.
GO:1901407; P:regulation of phosphorylation of RNA polymerase II C-terminal domain; IDA:UniProtKB.
GO:1901522; P:positive regulation of transcription from RNA polymerase II promoter involved in cellular response to chemical stimulus; TAS:BHF-UCL.
GO:1901581; P:negative regulation of telomeric RNA transcription from RNA pol II promoter; ISS:UniProtKB.
GO:1901582; P:positive regulation of telomeric RNA transcription from RNA pol II promoter; IMP:BHF-UCL.
GO:1901835; P:positive regulation of deadenylation-independent decapping of nuclear-transcribed mRNA; IDA:UniProtKB.
GO:1901837; P:negative regulation of transcription of nucleolar large rRNA by RNA polymerase I; IMP:UniProtKB.
GO:1901838; P:positive regulation of transcription of nucleolar large rRNA by RNA polymerase I; IMP:UniProtKB.
GO:1902064; P:regulation of transcription from RNA polymerase II promoter involved in spermatogenesis; ISS:UniProtKB.
GO:1902369; P:negative regulation of RNA catabolic process; IMP:UniProtKB.
GO:1902373; P:negative regulation of mRNA catabolic process; IMP:CAFA.
GO:1902415; P:regulation of mRNA binding; IEA:Ensembl.
GO:1902416; P:positive regulation of mRNA binding; IPI:ParkinsonsUK-UCL.
GO:1902629; P:regulation of mRNA stability involved in cellular response to UV; IMP:UniProtKB.
GO:1902679; P:negative regulation of RNA biosynthetic process; IEA:Ensembl.
GO:1902680; P:positive regulation of RNA biosynthetic process; IMP:ParkinsonsUK-UCL.
GO:1902894; P:negative regulation of pri-miRNA transcription by RNA polymerase II; ISS:BHF-UCL.
GO:1902895; P:positive regulation of pri-miRNA transcription by RNA polymerase II; ISS:BHF-UCL.
GO:1903025; P:regulation of RNA polymerase II regulatory region sequence-specific DNA binding; IMP:MGI.
GO:1903026; P:negative regulation of RNA polymerase II regulatory region sequence-specific DNA binding; IGI:GO_Central.
GO:1903632; P:positive regulation of aminoacyl-tRNA ligase activity; IEA:Ensembl.
GO:1903634; P:negative regulation of leucine-tRNA ligase activity; IEA:Ensembl.
GO:1903704; P:negative regulation of production of siRNA involved in RNA interference; IDA:BHF-UCL.
GO:1903798; P:regulation of production of miRNAs involved in gene silencing by miRNA; IEA:Ensembl.
GO:1903799; P:negative regulation of production of miRNAs involved in gene silencing by miRNA; IMP:BHF-UCL.
GO:1903800; P:positive regulation of production of miRNAs involved in gene silencing by miRNA; ISS:BHF-UCL.
GO:1903839; P:positive regulation of mRNA 3'-UTR binding; IDA:UniProtKB.
GO:1904582; P:positive regulation of intracellular mRNA localization; ISS:UniProtKB.
GO:1904812; P:rRNA acetylation involved in maturation of SSU-rRNA; IBA:GO_Central.
GO:1904872; P:regulation of telomerase RNA localization to Cajal body; IMP:BHF-UCL.
GO:1904874; P:positive regulation of telomerase RNA localization to Cajal body; IMP:BHF-UCL.
GO:1904911; P:negative regulation of establishment of RNA localization to telomere; IMP:BHF-UCL.
GO:1905216; P:positive regulation of RNA binding; IMP:CAFA.
GO:1905382; P:positive regulation of snRNA transcription by RNA polymerase II; ISS:UniProtKB.
GO:1905612; P:positive regulation of mRNA cap binding; IDA:ParkinsonsUK-UCL.
GO:1905618; P:positive regulation of miRNA mediated inhibition of translation; IDA:ParkinsonsUK-UCL.
GO:1905636; P:positive regulation of RNA polymerase II regulatory region sequence-specific DNA binding; IMP:ParkinsonsUK-UCL.
GO:1905662; P:negative regulation of telomerase RNA reverse transcriptase activity; IMP:BHF-UCL.
GO:1905663; P:positive regulation of telomerase RNA reverse transcriptase activity; ISS:BHF-UCL.

GO:1905869; P:negative regulation of 3'-UTR-mediated mRNA stabilization; IDA:UniProtKB.
GO:1905870; P:positive regulation of 3'-UTR-mediated mRNA stabilization; IMP:UniProtKB.
GO:1990074; P:polyuridylation-dependent mRNA catabolic process; ISS:UniProtKB.
GO:1990114; P:RNA polymerase II core complex assembly; IMP:UniProtKB.
GO:1990180; P:mitochondrial tRNA 3'-end processing; IDA:UniProtKB.
GO:1990247; F:N6-methyladenosine-containing RNA binding; IDA:UniProtKB.
GO:1990248; P:regulation of transcription from RNA polymerase II promoter in response to DNA damage; IDA:ARUK-UCL.
GO:1990261; P:pre-mRNA catabolic process; IMP:UniProtKB.
GO:1990269; F:RNA polymerase II C-terminal domain phosphoserine binding; IDA:UniProtKB.
GO:1990280; P:RNA localization to chromatin; ISS:UniProtKB.
GO:1990428; P:miRNA transport; ISS:BHF-UCL.
GO:1990440; P:positive regulation of transcription from RNA polymerase II promoter in response to endoplasmic reticulum stress; TAS:ParkinsonsUK-UCL.
GO:1990441; P:negative regulation of transcription from RNA polymerase II promoter in response to endoplasmic reticulum stress; IMP:ParkinsonsUK-UCL.
GO:1990481; P:mRNA pseudouridine synthesis; IMP:UniProtKB.
GO:1990511; P:piRNA biosynthetic process; ISS:UniProtKB.
GO:1990715; F:mRNA CDS binding; IEA:Ensembl.
GO:1990744; P:primary miRNA methylation; IDA:UniProtKB.
GO:1990817; F:RNA adenylyltransferase activity; IEA:InterPro.
GO:1990825; F:sequence-specific mRNA binding; ISS:UniProtKB.
GO:1990883; F:rRNA cytidine N-acetyltransferase activity; IBA:GO_Central.
GO:1990930; F:RNA N1-methyladenosine dioxygenase activity; IDA:UniProtKB.
GO:1990931; F:RNA N6-methyladenosine dioxygenase activity; IDA:UniProtKB.
GO:1990932; F:5.8S rRNA binding; IEA:Ensembl.
GO:1990968; P:modulation by host of RNA binding by virus; IMP:ParkinsonsUK-UCL.
GO:1990969; P:modulation by host of viral RNA-binding transcription factor activity; IGI:ParkinsonsUK-UCL.
GO:1990983; P:tRNA demethylation; IDA:UniProtKB.
GO:1990984; F:tRNA demethylase activity; IDA:UniProtKB.
GO:2000232; P:regulation of rRNA processing; IDA:UniProtKB.
GO:2000233; P:negative regulation of rRNA processing; ISS:UniProtKB.
GO:2000234; P:positive regulation of rRNA processing; IMP:UniProtKB.
GO:2000623; P:negative regulation of nuclear-transcribed mRNA catabolic process, nonsense-mediated decay; IMP:UniProtKB.
GO:2000626; P:negative regulation of miRNA catabolic process; IDA:UniProtKB.
GO:2000627; P:positive regulation of miRNA catabolic process; IDA:UniProtKB.
GO:2000628; P:regulation of miRNA metabolic process; ISS:UniProtKB.
GO:2000630; P:positive regulation of miRNA metabolic process; IMP:BHF-UCL.
GO:2000632; P:negative regulation of pre-miRNA processing; IDA:UniProtKB.
GO:2000637; P:positive regulation of gene silencing by miRNA; IMP:UniProtKB.
GO:2000721; P:positive regulation of transcription from RNA polymerase II promoter involved in smooth muscle cell differentiation; IEA:Ensembl.
GO:2000730; P:regulation of termination of RNA polymerase I transcription; IEA:Ensembl.
GO:2000763; P:positive regulation of transcription from RNA polymerase II promoter involved in norepinephrine biosynthetic process; ISS:BHF-UCL.
GO:2000805; P:negative regulation of termination of RNA polymerase II transcription, poly(A)-coupled; IDA:UniProtKB.
GO:2000806; P:positive regulation of termination of RNA polymerase II transcription, poly(A)-coupled; IMP:UniProtKB.
GO:2000815; P:regulation of mRNA stability involved in response to oxidative stress; IBA:GO_Central.
GO:2000820; P:negative regulation of transcription from RNA polymerase II promoter involved in smooth muscle cell differentiation; ISS:BHF-UCL.
GO:2000827; P:mitochondrial RNA surveillance; IMP:UniProtKB.
GO:2001141; P:regulation of RNA biosynthetic process; ISS:ARUK-UCL.
GO:2001165; P:positive regulation of phosphorylation of RNA polymerase II C-terminal domain serine 2 residues; IMP:CACAO.
